# Molecular Level Sucrose Quantification: A Critical Review

**DOI:** 10.3390/s22239511

**Published:** 2022-12-05

**Authors:** Gustavo A. Lara-Cruz, Andres Jaramillo-Botero

**Affiliations:** 1 Omicas Alliance, Pontificia Universidad Javeriana, Cali 760031, Colombia; 2 Chemistry and Chemical Engineering, California Institute of Technology, Pasadena, CA 91125, USA

**Keywords:** sucrose, neutral analyte, molecular recognition, low-molar mass analyte, biosensors, artificial receptors

## Abstract

Sucrose is a primary metabolite in plants, a source of energy, a source of carbon atoms for growth and development, and a regulator of biochemical processes. Most of the traditional analytical chemistry methods for sucrose quantification in plants require sample treatment (with consequent tissue destruction) and complex facilities, that do not allow real-time sucrose quantification at ultra-low concentrations (nM to pM range) under in vivo conditions, limiting our understanding of sucrose roles in plant physiology across different plant tissues and cellular compartments. Some of the above-mentioned problems may be circumvented with the use of bio-compatible ligands for molecular recognition of sucrose. Nevertheless, problems such as the signal-noise ratio, stability, and selectivity are some of the main challenges limiting the use of molecular recognition methods for the in vivo quantification of sucrose. In this review, we provide a critical analysis of the existing analytical chemistry tools, biosensors, and synthetic ligands, for sucrose quantification and discuss the most promising paths to improve upon its limits of detection. Our goal is to highlight the criteria design need for real-time, in vivo, highly sensitive and selective sucrose sensing capabilities to enable further our understanding of living organisms, the development of new plant breeding strategies for increased crop productivity and sustainability, and ultimately to contribute to the overarching need for food security.

## 1. Introduction

Global warming is a worldwide phenomenon that is the result of an accumulation of greenhouse gases in the Earth’s atmosphere [1]. Some of the consequences of global warming include changes in seasonal weather [2,3] and micro-climatic conditions [2,3], plant pathogen proliferation [4], and soil acidification [5]. These are imposing added stress conditions for crops and affecting food production for the world’s growing population [1,2,4,6]. The development of new agricultural crops capable of withstanding such stresses has become a necessity for food security and sustained productivity [1,2,4,6]. This can be achieved through the epigenetic and genetic improvement of plants [6,7,8,9], which requires a deep understanding of the underlying biochemical processes that drive stress response, and how these affect the genetic expression of an organism under a particular set of conditions. This includes elucidating key metabolic pathways that drive the production of biomass, from simple sugars to biomolecules [6,9].

Sucrose is a disaccharide that is composed of fructose and glucose. It is an end product of photosynthesis and it is the main sugar transported in the phloem of most plants [10,11,12]. Sucrose concentration is a biomarker for biochemical efficiency in plants because it is involved in numerous metabolic processes associated with growth and development, control, and signaling [11]. However, the mechanisms in which sucrose participates as a signaling molecule remain widely unknown [10,12]. The lack of chemical sensing techniques for quantifying sucrose under in vivo conditions exacerbates this problem [13,14]. Highly selective, sensitive, and spatiotemporally resolved techniques are needed [13]; and sucrose quantification under in vivo conditions may require sensing techniques with detection limits at nM concentrations, or below [13,14].

The usefulness of quantification techniques with a molecular resolution under in vivo conditions, and temporal resolution of seconds, is exemplified by the development and further application of genetically encoded nanosensors for glucose [15,16] and sucrose [14,16]. The codification and expression of genetically encoded nanosensors in mutants of oryza sativa allowed the identification of new SWEET proteins for the import of sucrose from mesophyll into phloem cells [17]. Genome edition, with CRISPR-Cas9, of these SWEET proteins allowed the development of oryza sativa varieties with improved resistance against bacterial blight disease in rice [18]. Nonetheless, techniques for in vivo quantification of sucrose with a high spatio-temporal resolution, are not only limited to elucidating the epigenetic mechanisms of sucrose. For example, they may allow monitoring concentrations of this analyte in real-time, speeding up the breeding of agricultural crops by identification of individuals with improved resistance against biotic or abiotic stresses, prior to the manifestation of phenotype differences [10,12,13]; or also may help in the early identification, and subsequent mitigation, of non-optimal concentrations of sucrose during plant development, fruit generation, seed development, or to detect the beginning of the flowering stage [10,11,12,19].

The main difficulties for ultra-low sucrose quantification in plants are selectivity, low molecular weight, high solvation, and electroneutrality at pH 7.4 [20]. Research and development for sucrose quantification have mainly been applied to food quality control assessments, which require high selectivity, albeit low detection limits are not imperative.

Here, we review the limits of detection (LOD), quantification (LOQ), and the range of detection achieved with conventional analytical chemistry techniques, biosensing, and artificial receptors sucrose quantification. In the first part of this review, we describe traditional analytical chemistry methods for sucrose quantification. Although this may overlap with Lowan’s earlier work (reported in 1978) on sucrose quantification in beets [21], and with a more recent review by Pokrzywnicka et al. on analytical methods for the determination of disaccharides [22], we provide a more comprehensive review of the analytical chemistry methods to establish a reference for comparison on new developments for sucrose quantification. The second part of this review focuses on biosensing and artificial receptors with potential application to sucrose quantification, based on the specific recognition of molecular shape, and covalent, or non-covalent interactions. We highlight the interactions, at the molecular scale, that have allowed the separation and identification of sucrose, and outline complementary criteria that may help in the design of new ligand molecules or separation protocols, and transduction processes that offer a high signal-noise ratio for sucrose quantification under in vivo conditions. This is especially critical for the design of selective synthetic receptors. We finalize this review by discussing the remaining challenges for sucrose quantification at the molecular level.

**Figure 1:**
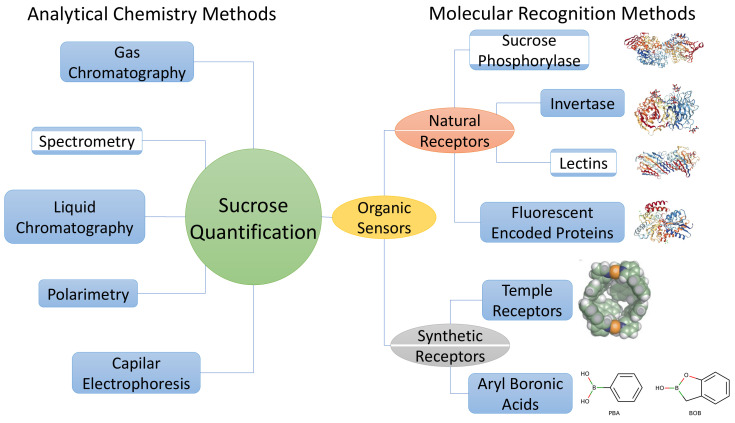
Overall sucrose detection and quantification techniques discussed in this review.

This paper is organized as follows: in Section 2, we present the physicochemical characteristics of sucrose as anchor variables for its quantification; in Section 3, we review traditional analytical techniques used currently for its quantification, such as polarimetry Section 3.1, liquid Section 3.2.1, gas chromatography Section 3.2.2, capillary electrophoresis Section 3.3, and spectroscopy Section 3.4. In Section 4, we highlight the molecular basis for selective sucrose quantification using biosensors Section 4.1, synthetic receptors Section 4.2, and molecular imprinted polymers Section 4.3. The topics covered are organized in the conceptual flow diagram shown in Figure 1.

## 2. Physicochemical Properties of Sucrose

**Figure 2:**
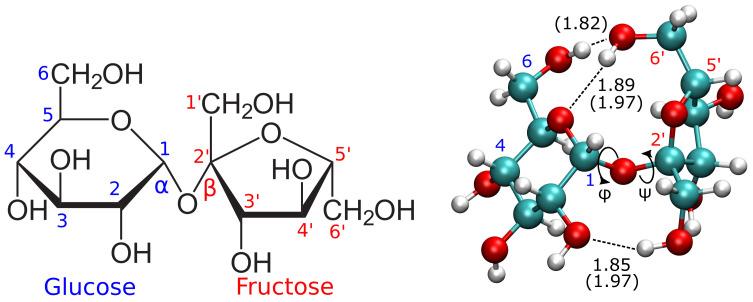
Haworth projection (**left**) and three dimensional atomistic representations of sucrose (**right**). The atomistic representations show the hydrogen bond distances from the crystalline structure [23] and from *ab initio* calculations (in parenthesis) [24].

Sucrose is composed of glucose and fructose with an α, β-glycosidic bond between the carbon atoms 1 and 2${}^{\prime }$ in glucose and fructose, respectively, as shown in Figure 2. From the crystalline structure, the dihedral angles for ϕ and ψ are 107.82${}^{\circ }$ and −44.75${}^{\circ }$, respectively, [23]. The most stable conformer of sucrose, calculated from DFT [24] and Molecular Mechanics [25] optimizations, have shown good coincidence with the molecular geometry of the crystalline structure. The dihedral angles of $\phi =111.4^{\circ }$ and $\psi =-52.7^{\circ }$ were reported from DFT calculations in a vacuum, while dihedrals of $\phi =104.5^{\circ }$ and $\psi =-54.9^{\circ }$ were reported from Molecular Mechanics optimizations with explicit water solvent molecules [25]. The most stable conformer of sucrose is favored by intramolecular hydrogen bonds (shown by dashed lines on the right side of Figure 2) and orbital hyperconjugation interactions across the atoms in the glycosidic bond [24]. Nonetheless, sucrose intramolecular hydrogen bonds are weakened in an aqueous solution, enabling conformational changes [25]. Molecular Mechanics simulations revealed mostly similar molecular geometries for sucrose in water, in comparison with its crystalline structure [25]. These were calculated from ϕ and ψ values during the molecular dynamics trajectories and validated with the experimental data from NMR spectra [25].

The α, β-glycosidic bond gives sucrose some of its chemical features [26,27]: a non-reducing sugar, without mutarotation process and no anomeric forms [27]; with a first dissociation constant of sucrose (pKa = 12.62) greater than the pKa of its parent carbohydrates (12.32 for glucose, and 12.02 for fructose) [28], which make sucrose a neutral molecule under physiological conditions. Table 1 shows some of the relevant physical properties of sucrose.

## 3. Analytical Chemistry Methods

### 3.1. Polarimetry

The interaction of a polarized monochromatic light beam with solutions of optically active organic compounds (For an organic compound to have optical activity, it must have at least one chiral carbon), rotates the polarization plane (${\left[\alpha \right]}_{\lambda }^{T}$) at the end of the optical path (*l*). This rotation of the polarization plane ${\left[\alpha \right]}_{\lambda }^{T}$ is inversely proportional to the concentration (*c*) of the optically active substance:

(1) $${\left[\alpha \right]}_{\lambda }^{T}=\frac{\alpha }{l\times c}$$

where α is the specific and characteristic rotation for each optically active compound [21].

Polarimetry is the most widely used method for the determination of sucrose in the industry [21]. Since polarimetry measures the rotation ${\left[\alpha \right]}_{\lambda }^{T}$ for all optically active substances in the sample solution, it is necessary to remove the optical activity of all substances other than sucrose to perform a single polarimetric measurement or perform sucrose inversion with the invertase enzyme (INV), and measure the change in polarization of the solution as sucrose grades [21].

Recently, Li et al. [29] reported a polarimetric method to measure sucrose inversion, in real-time, at concentrations range of μM. Instead of sodium D light, Li et al. used pulses of polarized light with a Gaussian distribution centered at 830 nm and amplitude of 10 nm, generated by a super luminescent diode (SLD) with a Gaussian filter. The reaction of sucrose with INV decreases the optical activity in the solution, changing the photon’s polarization at the optical interface and displacing the Gaussian center distribution of the photon wavelengths [29]. This is known as the photonic spin Hall effect due to the spin–orbit coupling in polarized photons. This spin–orbit coupling is a weak interaction that the authors managed to amplify under the approximation of quantum weak measurement, achieving the detection of sucrose in a linear range from 2.28 μM to 50.2 mM [29,30].

### 3.2. Chromatography

Chromatographic methods allow the detection and quantification of analytes when they are part of a complex mixture. The separation of the analytes is carried out by dissolving the sample in a proper solvent, liquid or gaseous, called the mobile phase. This solution flows through a solid, called the stationary phase, that interacts with the components of the sample. The interaction force between the analyte and the stationary phase depends on the chemical nature of each component in the mixture. Those that have a lower (higher) attractive force with the stationary phase have a shorter (longer) retention time and are the first (last) compounds eluted in the separation stage [22,31]. A detector is coupled to the separation phase to identify and/or quantify the analyte. This section covers the Liquid Chromatography (LC), capillary electrophoresis, and Gas Chromatography (GC) techniques used for sucrose quantification in different samples. Table 2 shows the LOD, LOQ, and the linear range of quantification achieved with these techniques.

#### 3.2.1. Liquid Chromatography

High-Performance Liquid Chromatography (HPLC) with a Refractive Index (RI) detector is the recommended technique for routine analysis of disaccharides in food [81]. However, new HPLC configurations for sucrose quantification are continuously reported with motivations such as improving the efficiency for the separation of the analyte in a specific sample and improving selectivity, sensitivity, and reproducibility. Some of these reports evaluate the performance of the normal phase [33,34,35,36] (the stationary phase is more polar than the mobile phase), reverse phase [37] (the mobile phase is more polar than the mobile phase stationary) and anion exchange columns [38,39,40,41,42,43,44,46,49] for sucrose quantification. Hydrophilic interaction liquid chromatography (HILIC) [48] has also been used to separate sucrose from mixtures with hydrophobic compounds. In normal phase configurations, columns with amino groups using a mixture of water-acetonitrile (40–95% acetonitrile) as mobile phase [22]. This water-acetonitrile mixture also is used in the reversed-phase configuration. One of the main advantages of HPLC is sucrose quantification without any derivatization.

In normal phase configuration, sucrose is an electrical neutral analyte; therefore, its separation and elution order depends on its polar affinity with the stationary phase. Conversely, in reverse phase configuration, sucrose is an anion analyte that interacts electrostatically with an ion-exchange resin column used as a stationary phase. This improves the sucrose separation from the sample and detection limits. Phase reverse configuration uses a strongly basic solution as the mobile phase (e.g., a solution of NaOH with a pH of around 11). Under these conditions, a significant amount of sucrose with the more acidic hydroxyl groups charged negatively is present in the solution [22].

Some of the detectors reported for sucrose quantification with HPLC are Evaporative Light Scattering Dispersion (ELSD) [33,34,49,50], Pulsed Amperometric Detection (PAD) [38,39,41,42], RI [36,37,43], Charged Aerosol detector (CAD) [34,35], and mass spectrometer (MS) [40,44,45,46,47,48]. The RI detector has the advantage of being fast and inexpensive, at the expense of low sensitivity. This can be improved by the use of ELSD or MS detectors that requires solvent evaporation. The PAD detector, used in anion exchange chromatography, also has better sensibility than the RI detector for sucrose quantification. Sucrose oxidation can be tuned given specific pH and voltage conditions in the PAD detector [22]. To conclude, the best results for sucrose quantification in plant samples were reported by Sevcik et al. [41]. The authors used an HPLC configuration with a modified anionic interchange column and a PAD detector; achieving LOD and LOQ of 3.5 nM and 11.3 nM, respectively, and linear detection range from 64.2 nM to 502.4 nM [41].

#### 3.2.2. Gas Chromatography

Gas chromatography has also been used for the quantification of sucrose in samples of plants [50,52], soils and sediments [51]. For gas chromatography, nitrogen as a gas carrier should be used for Flame Ionization Detector (FID); or Helium if a mass spectrometer is used as a detector. Contrary to LC which requires mobile phase optimization and the use of ultra-pure solvents, the use of gas carriers for GC avoids the need for optimizing the mobile phase. Additionally, the greater number of theoretical plates for GC improves the resolution of the mixture [52].

Quantification of sucrose by GC usually requires injector inlets and transfer line temperatures around 300 ${}^{\circ }$C, and oven gradient temperatures from 60 ${}^{\circ }$C to 300 ${}^{\circ }$C for the analyte separation [50,51]. However, these temperatures are well above sucrose degradation temperature (from 168 ${}^{\circ }$C to 189 ${}^{\circ }$C [27]); making it necessary to derivatize sucrose to improve its thermal stability. This derivatization process is the main difficulty for the application of GC because it is a complex process with long reaction times, and it must be carried out before the mixture resolution in the chromatographic column. The thermal stability of sucrose increases after reaction with aldononitrile acetate [50], *O*-methoxyoxime-trimethylsilyl [50,52] and trimethylsilyl [51] to form sucrose derivatives. These chemical modifications reduce the polarization of sucrose, improving its volatility and ionization efficiency [22,50,82]. The best performance for sucrose quantification in plant samples, by means of GC, was reported by Cai et al. [52]. These authors reported the quantification of sucrose, in fluids of tobacco samples, with LOD of 0.26 μM and linear detection range in the μM order using an FID detector and derivatization of sucrose with *O*-methoxyoxime-trimethylsilyl method [52].

### 3.3. Capilar Electrophoresis

Capillary electrophoresis (CE) is an analytical technique that separates the components of a mixture, based on the differences in the electrophoretic mobilities. The electrophoretic mobility of a single component is directly proportional to the charge of the analyte and inversely proportional to the viscosity of the solvent and the diameter of the atoms [31]. In order to separate the analyte, a small volume of the mixture (in the range of μL to nL) flows into a buffer-filled fused-silica capillary, and an applied electric field exerts a Coulomb electrostatic force on all the charged species of the mixture [31]. The dimensions of the capillary for CE are typically 10 to 100 mm in internal diameter and 40 to 100 cm long [22].

Neutral analytes can not be quantified with CE. Therefore, quantification of sucrose with CE requires basic pH buffers to charge the hydroxyl groups negatively (similar to ion exchange chromatography) [22]. The detection of sucrose after the electrophoretic separation has been performed in other studies by electrochemical and spectrometric measures. The electrochemical measures consist of the oxidation of the hydroxyl groups of the sucrose in basic conditions, for which a PAD detector is used [58,59,60,61,62]. Sucrose lacks any stronger activity in the UV region, which is the reason why two alternatives have been used for sucrose spectrometric measurements; (1) the derivatization of sucrose for direct detection with UV [55], (2) indirect detection by the use of chromophores in the buffer solutions such as sorbate [54], maleic acid, or phthalic acid [53]. The chromophores in the buffer solution can be detected with a Diode Array Detector (DAD) [53,54,55,56] or Chemiluminescence (CL) measures [57].

CE has achieved sucrose quantification with performance comparable to HPLC and GC techniques, regardless of simpler and cheaper instrumentation compared to HPLC and GC. Some authors have reported sucrose quantification using CE with LOD of 0.26 μM [59], and LOQ and linear detection ranges in the μM using amperometric detectors [61,62].

### 3.4. Spectrometry

Infrared spectroscopy (IR) is another analytical technique used to quantify sucrose. Sucrose quantification by IR spectroscopy is based on the simultaneous detection of the excited vibrational modes of the sucrose, after the interaction of the sample with IR photons. Table 3 shows some of these normal vibrational modes used for sucrose quantification with IR spectroscopy. Nonetheless, multivariate statistical models such as Partial Least Squares (PLS) are used to differentiate the signals of the normal modes of sucrose, from the normal modes of other molecules (including other sugars) with the same chemical groups of sucrose.

PLS is a predictive model for data with high collinearity. The construction of a PLS model requires a Principal Component Analysis (PCA) to identify the most important vibrational frequencies of the data set, with variations proportional to the sucrose concentration [63]. Some authors have performed a pre-processing of the data set to establish a baseline for the quantification of sucrose; or they have reduced the signal/noise ratio of the data by calculation of the first derivative, second derivative, normalization, smoothing, or the use of mean center functions [22,64]. Some authors have used the full IR spectrum (200–2000 cm${}^{-1}$) for the construction of PCA models [64], or have carried out the pre-treatment of the spectra at some specific frequencies of the sucrose, as reported in Table 3. The use of the PLS model was applied to quantify sucrose in complex samples (mostly food) such as lemon-type soft drinks [63], commercial soft drinks [64], solutions with fructose and glucose [65], sugar cane juice [83], bayberry juice [84], and sugar fruit juices [66], among others.

Nuclear Magnetic Resonance (NMR) is also a spectroscopic technique used for sucrose quantification by means of the diffusion ordered-quantitative ${}^{1}$H-NMR spectroscopy (DOSY-qNMR) [67]. The measurements carried out with DOSY-qNMR are based on the diffusion coefficient for different molecular species. Cao et al. [67] used the characteristic diffusion coefficient D of the sucrose to find a linear relationship between the sucrose concentration and the resonance area of the hydrogen atom bonded in the position glucopyranosyl-α-C1 of the sucrose. This linear relationship is valid in concentrations ranging from 1.46 mM to 58.42 mM, allowing the authors to differentiate sucrose from fructose, glucose, and cellobiose in orange juice, pineapple juice, and a sports drink [67].

The spectroscopic measurements do not require sample pre-treatment or derivatization. Instead, they require a calibration that strongly depends on the sample matrix. Therefore, a calibration must be performed for each type of sample, and sometimes a calibration curve for each component of the sample [22]. This is one of the reasons why detection ranges for spectroscopic methods in Table 2 show differences from mM to M order (three orders of magnitude, despite the measures being performed for samples from a liquid matrix [63,64,65,66,67].

### 3.5. Summary of Analytical Chemistry Methods

Analytical chemistry methods mentioned above are well-established techniques, with a well-understood physicochemical background for sucrose quantification. HPLC has a high-resolution capacity for complex carbohydrate mixtures, enabling the detection and quantification of sucrose in a mixture of carbohydrate isomers. LODs, LOQs, and detection range in the μM, are achieved with routine configurations for HPLC. Sucrose detection in the nM range can be achieved with proper sample preparation and HPLC settings. Nevertheless, this requires highly trained and experienced personnel. In addition, analytical chemistry measures are expensive and require complex laboratory facilities and large amounts of reactants. Sucrose quantification with GC shows similar performance to HPLC for routine configurations, with detection ranges in the μM order. Although optimization of the mobile phase is simpler for GC than HPLC, the complexity of the derivatization of sucrose biases the preference for HPLC over GC for sucrose quantification. Additionally, HPLC allows the quantification of other sugars than sucrose in the same run.

Although the CE is simple, uses low-cost instrumentation, and is low in solvent consumption, it has achieved similar performance to HPLC for sucrose quantification. The simplicity of CE instrumentation facilitates the miniaturization of CE instruments [22] for use in sucrose quantification in the field. Probably, the development of reliable miniaturized CE instruments may replace soon the use of polarimetry for routine quantification of sucrose in the field. Nevertheless, sucrose quantification with CE is a complex procedure that requires highly trained personnel to optimize the mobile for the electrophoretic separation of the analytes.

None of the analytical methods, except spectroscopy, allow the quantification of sucrose under in vivo conditions due to sample treatment for analyte separation. No sample pretreatment and coupling of IR spectrometers to microscopes have allowed the identification of carbohydrates in botanical samples [85,86]. However, the LOD for carbohydrates in botanical samples allows only a semiquantitative distribution of sucrose in plant tissues. Further investigations on IR and Raman spectroscopy, applied to in vivo quantification of sucrose, may be directed to the improvement of chemometric protocols by means of artificial neural networks, convolutional neuronal networks, or Deep Learning [87,88,89]. Although based on the detection ranges for liquid samples (see Table 2), the LOD for sucrose quantification with spectroscopic techniques, in botanical samples, may be limited to concentrations in the mM range.

## 4. Sucrose Quantification Using Molecular Recognition Methods

In this section, we review the main characteristics of molecular receptors, natural and synthetic, capable of selectively binding sucrose. Enzymes and proteins include: invertase (INV) [68,69,70,71,72,73,74], sucrose phosphorylase (SP) [75,76], Fluorescent Indicator Proteins (FLIP) [14,90], and lectins [91,92,93,94,95].

### 4.1. Natural Receptors

**Figure 3:**
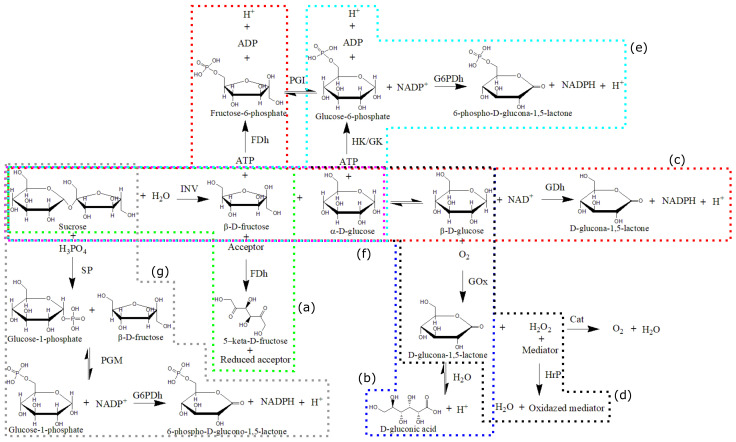
Sequence of enzymes for cascade reactions in enzymatic biosensing of sucrose: (**a**) INV → FDh (green box) [68]; (**b**) INV → Mut → GOx (blue box) [69,72]; (**c**) INV → Mut → GDh and INV → FDh (red box) [70]; (**d**) INV → Mut → GOx → HrP (black box) [71]; (**e**) INV → HK/GK → G6PDh (cyan box) [73]; (**f**) INV (magenta box) [74]; and (**g**) SP → PGM → G6PDh (grey box) [75,76]. Figure adapted from reference [22]. Reprinted by permission from the publisher Taylor & Francis Ltd., London, England. http://www.tandfonline.com (accessed on 1 October 2022).

We start this part of the review with the INV and SP enzymes, commonly used in cascaded bio-sensing devices. In cascaded biosensors, the INV and SP enzymes are used to sense sucrose, selectively, as the initial step of a series of sequential reactions. The products from a step reaction are used as substrate in the next step of the sequence of reactions of the known stoichiometry until the appropriate molecule for detection is produced in the last step reaction [22]. This allows the direct quantification of sucrose in a complex sample. Figure 3 shows the typical reaction sequences used in sucrose biosensors. For INV, the first step reaction transforms sucrose to glucose and fructose [68,69,70,71,72,73,74]. For SP, the first step reaction transforms sucrose to glucose 1-phosphate and fructose [75,76].

We continue the review with recent developments on fluorescent indicator protein (FLIPs); a non-destructive quantification method for sucrose based on conformational changes of the protein after sucrose binding, and intensity changes in the Föster Resonance Energy Transfer (FRET) process [14,90]. FLIPs have been successfully used for the in vivo quantification of glucose and sucrose [16]. Finally, we close this section by reviewing the structural features of lectins [91,92,93], a group of proteins widely present in living organisms but not used for sucrose (or carbohydrate) biosensors due to their poor selectivity and lack of signal transduction after binding to sucrose [91,92,93,94,95].

#### 4.1.1. Invertase (INV)

**Figure 4:**
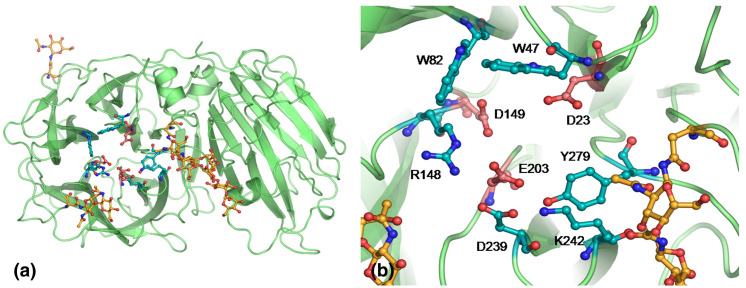
(**a**) AtcwINV1 invertase enzyme helix and sheet representation. (**b**) Close-up view of the active site for AtcwINV1. The conserved residues of D23, D149 and E203 are shown in red. The figure is taken from reference [98]. Reprinted from the *Journal of Molecular Biology*, 377, Willem Lammens et al., Crystal Structures of Arabidopsis thaliana Cell-Wall Invertase Mutants in Complex with Sucrose, 378–385, Copyright (2008), with permission from Elsevier.

**Figure 5:**
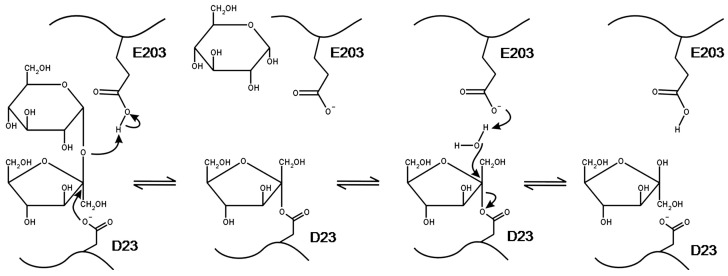
Reaction mechanism for INV enzymes. D23 and E203 are identified as nucleophile and acid/base catalysts, respectively. The figure is taken from reference [98]. Reprinted from the *Journal of Molecular Biology*, 377, Willem Lammens et al., Crystal Structures of Arabidopsis thaliana Cell-Wall Invertase Mutants in Complex with Sucrose, 378–385, Copyright (2008), with permission from Elsevier.

INV is part of the glycoside hydrolases GH32 family, present in plants and microorganisms. This protein family has six conserved motifs (A–F) within the N-terminal domain, in which the motifs A, D, and E contain the active site. From the Hidden Markov Model (HMM), conserved sequences for motifs around the active site are WMN**D**PNG (for motif A), FR**D**P (for motif D), and MW**E**CPDF (for motif E). Bold letters correspond to the active site residues [96,97]. For INV, residues in the active site can be enumerated as D23, D149 and E203 (see Figure 4), forming a catalytic triad with specific roles for sucrose hydrolysis, as shown in Figure 5 [98,99]. D23 is the catalytic nucleophile that bonds to the anomeric carbon in fructose. E203 is the acid/base catalyzer that stabilizes the intermediates in the glycosidic bond cleavage (with E203 as proton donor), and the deglycosylation step (with E203 as proton receptor). D149 provides key hydrogens for sucrose recognition, bonding the C3’ and C4’ hydroxyls in fructose to the active site [97,98,99].

INVs are classified as acid, neutral, or basic according to their optimal pH activity. Acid INVs have an optimal pH activity between 4.5 and 5.0 (an apparent Michaelis-Menten constant activity $K_{m}$ in the low mM range) and are inhibited by heavy metal ions (such as Hg${}^{2+}$ and Ag${}^{+}$), by glucose (as a non-competitive inhibitor), and by fructose (as a competitive inhibitor) [100,101]. On the other hand, neutral and basic INVs have an optimal pH activity between 7.0 and 7.8 with a $K_{m}$ of 10 mM, hydrolyzing only sucrose. Neutral and basic INVs are not inhibited by metal ions, but they are strongly inhibited by glucose, fructose and Tris [100,101].

The recombinant INV from *Saccharomyces* (*S*INV) is an octameric enzyme. The crystal structure shows at least two catalytic pockets of different sizes and environments determined by the quaternary structure [97]. One of the catalytic pockets, with dimensions of 10 ×10 Å, seems unable to host carbohydrates with no more than three or four sugar units. The other pocket has dimensions of 20 × 16 Å and might host polysaccharides, but this implies a high energy distortion penalty. These catalytic pockets are rigid structures because most of their amino acids are short-chain amino acids. Size and rigidity of the *S*INV pockets make this enzyme highly selective to sucrose instead of trisaccharides, tetrasaccharides, or inulin (see Table 4) [97].

Vargas et al. [68] have reported the best performance for sucrose quantification using an INV biosensor. The first reaction step in the Vargas biosensor is the hydrolysis of sucrose by INV to produce β-D-fructose. Then, β-D-fructose reacts with the FDh enzyme for oxidation to 5-keto-D-fructose. This last reaction is a RedOx reaction, allowing the authors to quantify sucrose using an amperometric detector. Vargas et al. [68] reported an LOD of 0.36 μM for sucrose, and linear detection ranges from 1.2 μM to 3.0 mM [68].

#### 4.1.2. Sucrose Phosphorylase (SP)

SP is an enzyme present in a small number of bacterial species, classified into the glycoside hydrolases family GH13_18 [102]. SP catalyzes the reversible transglucosylation of sucrose and phosphate (HPO${}_{4}^{2-}$ or H${}_{2}$PO${}_{4}^{-}$) into fructose and glucose-1-phosphate, with the equilibrium condition biasing to the transglucosylated products [102,103].

**Figure 6:**
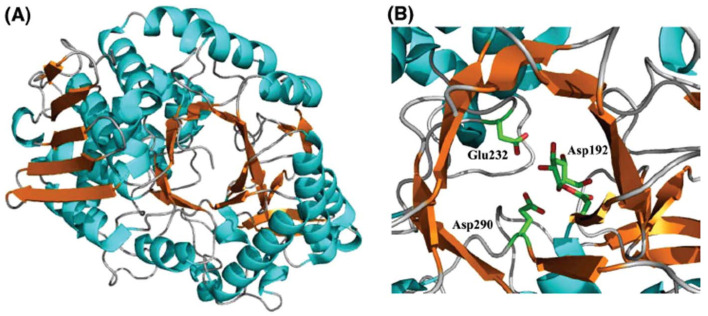
(**A**) Helix and sheet representation for a monomer unit of SP from *B. adolescentis*. Crystal structure reported in PDB entry 1r7a. (**B**) Close-up view of the active site of SP from *B. adolescentis* (PDB entry 2gdv, chain a). The residues highlighted belong to the catalytic triad Asp192 (glucosylated in the structure), Glu232 and Asp290. Figure from reference [102]. Reprinted by permission from the publisher Taylor & Francis Ltd., London, England. http://www.tandfonline.com (accessed on 1 October 2022).

**Figure 7:**
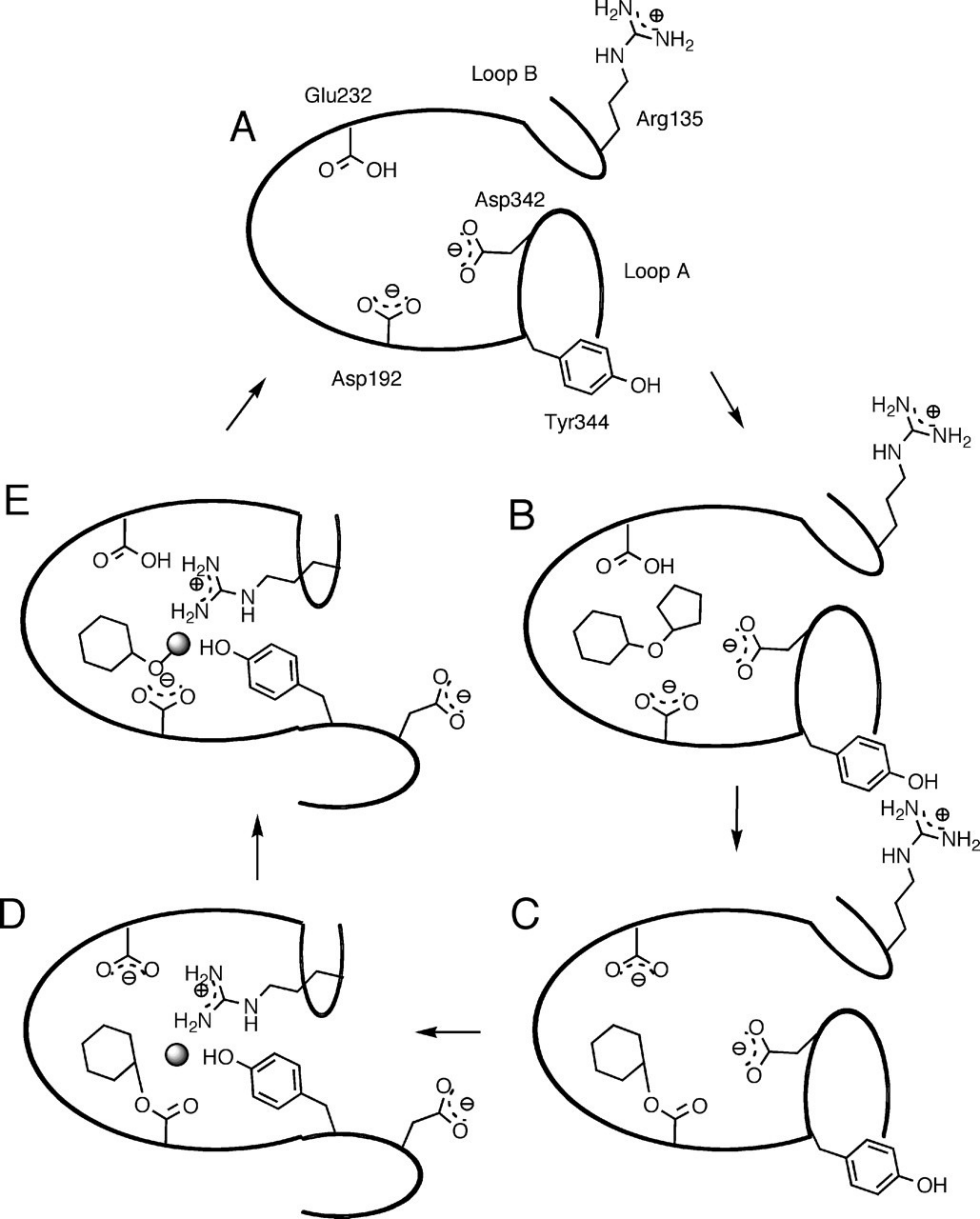
Reaction mechanism scheme for sucrose transglucosylation in the active site of a model SP enzyme. Step (**A**) corresponds to the apoenzyme, which binds the substrate sucrose in step (**B**), to form the covalent intermediate in step (**C**), with fructose leaving the active site. In step (**D**), the glucosyl intermediate reacts with either phosphate ions (phosphorolysis) or water (hydrolysis). Phosphate/water is depicted as a gray sphere. Step (**E**) shows the product-bound form of the enzyme (hydrolysis, glucose; and phosphorolysis, glucose 1-phosphate). Only the carboxylate groups of Glu232 and Asp192 are shown for clarity. The glucosyl and fructosyl groups are represented by hexagons and pentagons, respectively. Figure from reference [105].

Figure 6 shows a cartoon representation of the SP enzyme extracted from the *Bifidobacterium adolescentis* (*Bi*SP) bacteria [103]. The crystal structure reveals a four domains protein (namely A, B, B’, and C), where the active site is localized at the tips of β-sheets 4 and 5 in the (β/α)${}_{8}$-barrel, as common for the members of the GH13 family [103]. Similar to INV, two aspartates (D192 and D290) and one glutamate (E232) are the most important amino acids for the transglucosylation of sucrose by SP. D192 is the catalytic nucleophile, and E232 is the catalytic acid/base. D290 has the roles of sucrose recognition and stabilization of the transition state, forming hydrogen bonds with the hydroxyl groups of C2 and C3 in the glucose ring [102]. Figure 7 shows the catalytic cycle of SP, where the double displacement reaction for transglucosylation of sucrose, occurs concurrently with conformational changes of the protein [104]. Some researchers [102] had related the SP flexibility to its capacity to produce a variety of transglucosylated of sucrose products with glycerol, arabinose, xylitol, and others [102].

Geometrical features of SP control its chemical activity and selectivity towards sucrose. A loop in the B domain tune the size channel entry to the active site, biasing the reactivity of SP towards sucrose or saccharides of similar size, excluding polysaccharides to reach the active site [105]. $K_{M}$ reported for SPs are in the range of 1–15 mM [104], with optimal pH conditions from 6.0 to 7.0 and temperatures from 30 ${}^{\circ }$C to 48 ${}^{\circ }$C [102] for transglucosylation. It was found that glucose, fructose, and phosphate could inhibit the SP from *P. saccharophila*, with an affinity for glucose 500 times greater than fructose or phosphate [103].

Kogure et al. [76] used a cascade biosensor based on SP for the selective sucrose quantification on five soft drink samples. This biosensor transformed sucrose until NADPH (see Figure 3) for detection of this last molecule by spectro-fluorimetry [76], achieving a LOD of 0.1 μM and linear detection to 200.0 μM [76].

In general, sucrose biosensors based on the INV and SP enzymes may have, typically, LOD in the mM order. Although it is noteworthy to highlight the results in the references [68,72,76], where the authors reported detection ranges in the μM for cascade biosensors. Despite the high selectivity of the INV and SP enzymes for their reaction with sucrose, these enzymes require specific conditions for their operation and storage to avoid their degradation, limiting their use to controlled conditions. In addition, enzyme immobilization is an expensive and time-costly step for biosensor assembling [106]. An important factor that forbids the use by INV and SP biosensors for in vivo quantification of sucrose is the hydrolysis reaction by these enzymes. Sucrose hydrolysis will alter the central metabolism of the plant, modifying the homeostatic conditions in the plant and inducing a fake stress condition.

#### 4.1.3. Fluorescent Indicator Proteins (FLIPs)

**Figure 8:**
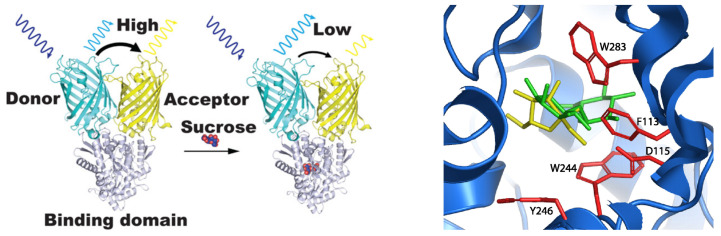
(**Left**) Model representation for the FLIPsuc-$\Delta^{1eCFP-eYFP}$ sensor in free form and bonded to sucrose [90]. Sucrose binding triggers conformational rearrange of sugar-binding protein, quenching the emission fluorescence from the FRET process [14]. (**Right**) Close-up view of the carbohydrate binding site in FLIPsuc-$\Delta^{1eCFP-eYFP}$ chimeric protein. In red are the residues key for carbohydrate binding, yellow is for the sucrose representation, and green is for maltose representation. Left figure taken from the reference [14], and the right panel figure taken from the reference [90].

Fluorescent Indicator Proteins (FLIPs) are an example of non-destructive biosensors used for sucrose quantification [14,90]. These biosensors use as a scaffold a sugar-binding protein (SBPs) from *Agrobacterium*, functionalized with two fluorescent proteins; the cyan (eCFP) and the yellow fluorescent protein (eYFP). Each fluorescent protein was attached to the N and C termini of the SBP, producing the chimeric protein FLIPsuc-4μ[90]. The eCFP and eYPF proteins act as a FRET, from eCFP to eYPF (see Figure 8). The binding of sucrose to the FLIPsuc-4μ induces a conformational change in the protein, reducing the efficiency of the FRET processes and quenching the fluorescent emission by the eYPF protein. The fluorescence quenching is directly proportional to sucrose quantification.

FLIPsuc-4μ has a dissociation constant $K_{d}=3.7$μM for sucrose, but a higher affinity for maltose ($K_{d}=0.23$μM) [90]. A series of modifications residues in the FLIPsuc-4μ binding site (namely F113A, W283A, D115A, D115E, Y246A, and W244A) allowed the identification of mutation W283A, being key to bias FLIPsuc-4μ selectivity 50 times towards sucrose over maltose, 10 times over glucose, but reducing the sucrose affinity to $K_{d}$ = 88 μM. Loss of stacking interactions between sucrose and the tryptophan residue 283, by mutation with alanine, is the main reason for the reducing affinity [90]. Although, this mutation improves selectivity towards sucrose; because alanine mutation removes the steric clash of tryptophan 283 with fructose moiety of sucrose [90].

Further improvement of FLIPsuc by optimization of pair FRET transducers, linker length, and residue mutation near the binding site. This optimization improved the FLIPsuc detection range from 7 μM to 585 μM, the signal/noise ratio for FRET transduction, and the affinity for sucrose to $K_{d}=65$μM without any loss of selectivity [14].

Successful gene codification and expression of FLIPs in plants have allowed the identification of new SWEET transporters involved in the carbohydrate uptake in roots [15], sucrose transport into phloem cells [60], translocation of sucrose into seeds [107], production of floral nectar [108], and the resistance against pathogens [18]. Nevertheless, some FLIP variants did not show any FRET signal in cytoplasmic conditions [15]. Furthermore, signal transduction in sucrose FLIPs by FRET appears to be limited to LOD of μM concentrations [14], which may be insufficient to quantify some biochemical processes where sugar concentrations have been below 90 nM [15].

#### 4.1.4. Lectins

Lectins are carbohydrate-binding proteins ubiquitous present in nature with wide biological functions such as molecular recognition, cell adhesion, and regulation of the biochemical process, among others [109]; lack catalytic activity and bind reversibly to carbohydrates, ranging from monosaccharides to polysaccharides, present in solution or glycoproteins [109]. Lectins are oligomeric proteins that are classified according to the monosaccharide for which present the highest affinity: mannose/glucose, galactose/N-acetylgalactosamine, N-acetylglucosamine, fucose, and N-acetylneuraminic acid [109].

Legume lectins were used to elucidate the basis for carbohydrate recognition of lectins [93]. The carbohydrate-binding site (CBS) of legume lectins is located in the concave face of a seven-stranded β-sheet [91]. Legume lectin CBS is a shallow pocket, formed by five motifs with a highly conserved triad of amino acids (asparagine, aspartate, glycine, or arginine). These amino acids recognize carbohydrates by hydrogen bonds, while hydrophobic residues such as phenylalanine, tyrosine, tryptophan, or leucine, stabilize the carbohydrate by stacking (hydrophobic) interactions [91,110]. Two divalent cations, a Ca${}^{2+}$ into the CBS, and an Mn${}^{2+}$ adjacent to the CBS, fix the spatial disposition of amino acids for carbohydrate binding [91,111]. Although legume lectins have highly conserved secondary and tertiary structures, they have a wide variety of quaternary structures, which are related to the wide range of carbohydrate affinities among lectins [92,109].

**Figure 9:**
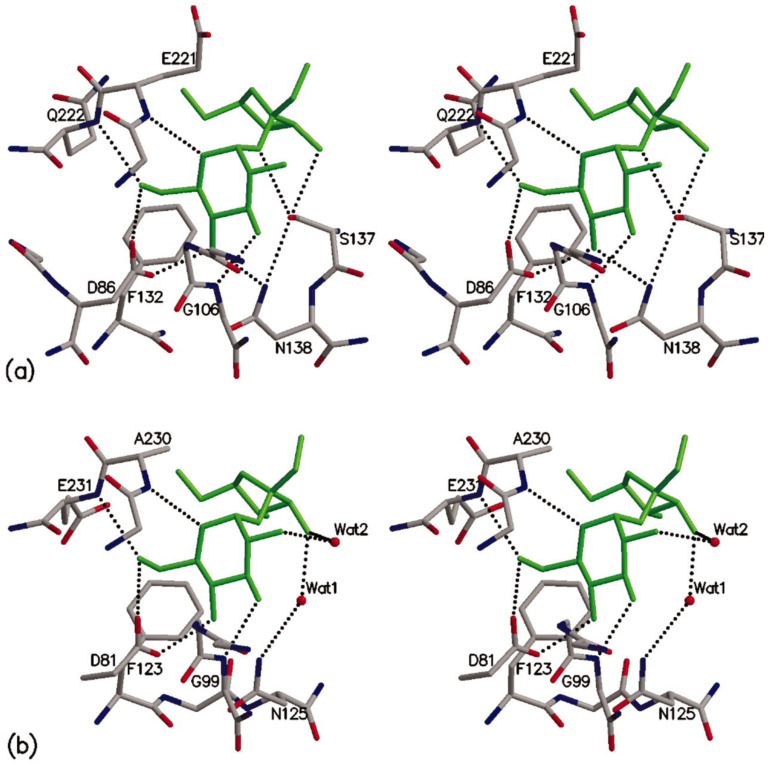
Binding sites of sucrose in PAL and lentil lectin. (**a**) omplex of sucrose with PAL. (**b**) equivalent complex with lentil lectin shown in the same orientation. Figure taken from reference [93].

Figure 9 shows the crystal structure of sucrose with lentil lectin [92], and *Pterocarpus angolensis* lectin. Sucrose is bound to the CBS by the glucosyl side, with an aspartate (D86) accepting hydrogen bonds from -OH groups in C4 and C6. The nitrogen atoms in asparagine (N138) and peptide NH of glycine (G106) act as donors in the hydrogen bonds to -OH groups in C4 and C3, respectively, [91]. Stacking interactions are established between the hydrophobic face of glucosyl moiety (equatorial H in C2, and axial Hs in C3 and C5) and phenylalanine (F123) residue [91,92,93]. The fructosyl moiety does not form any direct hydrogen bond with CBS in lentil lectin [92], while in *Pterocarpus angolensis* lectin a direct hydrogen bond is formed with serine (S137) [93].

The affinity of lectins to monosaccharides is usually weak, with association constants $K_{b}$ to monosaccharides typically ranging from 103 to 500 M ${}^{-1}$ [94,95], whereas that to oligosaccharides is often much higher, up to three orders of magnitude [111].Probably, the absence of lectins biosensors is related to the low binding coefficients of lectins to sucrose (and other carbohydrates) [94,95]. Additionally, lectins do not show any structural change when complexed with sucrose. Then, the signal transduction by the lectin-sucrose binding is hard to detect.

### 4.2. Synthetic Receptors

Biosensors allow sucrose quantification with a performance similar to the analytical chemistry methods. However, biosensors have several limitations due to the need for protein preservation, limited lifetimes, specific reaction conditions, and high cost for protein immobilization, among others [112]. Synthetic receptors are an alternative for sucrose sensing that can overcome some of the biosensor limitations [94,113,114,115,116,117,118,119,120,121]. In this paper, we reviewed the results of temple receptors (Section 4.2.1) and ligands based on aryl boronic acid (Section 4.2.2), applied for the selective binding of sucrose. Although it is worth mentioning, some of the developments in synthetic receptors have been motivated, mainly, by selective glucose quantification [94,115], and the results for sucrose binding have been part of the screening process for selectivity among carbohydrates.

Table 5 provides a summary of the monosaccharides and disaccharides that have been detected using natural or artificial receptors.

#### 4.2.1. Temple Receptors

Temple receptors are molecules designed to host (or encapsulate) carbohydrates inside a cavity, which is formed by assembling polyaromatic molecules acting as ceiling and floor, and defining the length of the cavity [114,115,116]. Molecules with either amino [114,116] or hydroxyl [115] groups act as pillars, defining the height of the cavity. Carbohydrates interact with the temple through non-covalent interactions in the cavity; only are hosted by temple receptors, carbohydrates with size and shape concomitant with the cavity dimensions [113,114,115,116].

**Figure 10:**
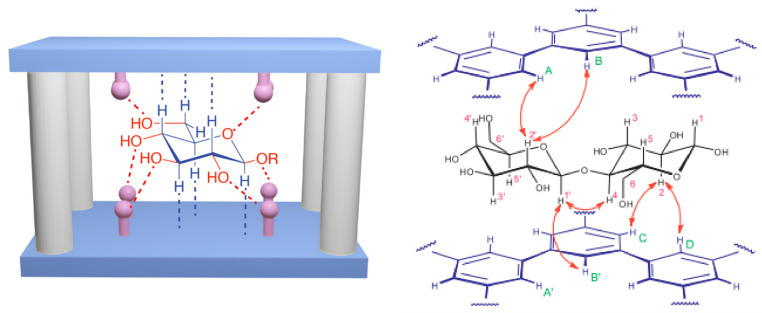
(**Left**) Temple receptor scheme for the glucose binding with C-H⋯π, C-H⋯O and N-H⋯O non-covalent interactions. The red dashed lines represent the polar interactions and the blue ones the hydrophobic interactions. (**Right**) Roof and floor components of the temple type receptor for the D-Cellobiose disaccharide. The left figure was reprinted with permission from [115]. Copyright (2021) American Chemical Society. The right figure was taken from reference [114], and reprinted with permission from AAAS.

Tromans et al. [95] reported temple receptors with high selectivity towards glucose, with a binding coefficient of $K_{b}$ of 18,200 M${}^{-1}$ for this monosaccharide, and $K_{b}$ one hundred times smaller for other carbohydrates. These differences between the $K_{b}$s have allowed complete exclusion of carbohydrates interferents in glucose quantification [95]. The left side in Figure 10 shows the interactions between glucose and a model temple receptors [114,115,116]. The hydrogens at the axial position interact with the π electrons of the roof and floor molecules, and the hydroxyl groups at the equatorial positions interact with the pillars of the cavity through hydrogen bonds. The right panel of Figure 10 shows the roof and floor molecules of a temple receptor designed for selective binding with the disaccharide D-Cellobiose, and its derivatives [114]. The authors reported a $K_{b}$ of 910 M ${}^{-1}$ for β-D-cellobioside, and a $K_{b}$ of 580 M${}^{-1}$ for D-cellobiose [114].

**Figure 11:**
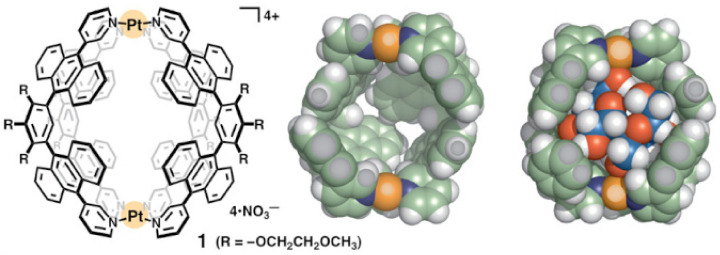
(**Left**) Molecular representation and (**Middle**) its space-filling representation for the nanocapsule based on Pd(II) and anthracene for selective sucrose binding. (**Right**) corresponds to the complex sucrose-nanocapsule in space-filling representation. Substituents and counterions are omitted for clarity. Figure from reference [122].

Carbohydrates with β-glucopyranoside structures, lying in the same molecular plane, are the most suitable carbohydrates for complexation with temple receptors of cubic cavity shape [114]. Sucrose differs from the structural features above mentioned, and, probably, this is the main reason why no binding has been detected between sucrose and temple receptors [130]. This conclusion is reinforced by the work of Yamashina et al. [122], in which the authors reported a nanocapsule that binds sucrose, in water, with a selectivity of ∼100 %, excluding other natural disaccharides such as D-lactose, D-maltose, D-cellobiose, and D-lactulose [122]. Figure 11 shows this nanocapsule; a coordination complex of stoichiometry M${}_{2}$L${}_{4}$ formed Pt(II) and polyaromatic anthracene panels. The authors reported a $K_{b}\geq 1100$ M${}^{-1}$ for the nanocapsule-sucrose binding. Sucrose is bound into the nanocapsule through stacking interactions (no hydrogen bonds are formed). Complementarity of size and shape, between sucrose and the cavity of the nanocapsule, seems to be the principal factor for the high selectivity. The nanocapsule cavity has dimensions of ∼1 nm diameter and ∼580 Å${}^{3}$, and sucrose dimensions are ∼1 nm length and ∼300 Å${}^{3}$) [122].

Quantification of low sucrose concentrations remains an open challenge for temple receptors because they have not achieved a high binding coefficient $K_{b}$. Although the nanocapsule of Figure 11[122] sheds light on the geometrical shape and dimensions of the cavity for the high selectivity of sucrose, the formation of hydrogen bonds between sucrose and the host may be necessary to achieve high binding constants $K_{b}$. Another problem of temple receptors is the lack of signal transduction after the carbohydrate complexation. The fluorescence spectra of temple receptors do not change after the carbohydrate-binding event. For instance, the binding coefficient $K_{b}$ for the nanocapsule-sucrose complex was measured by NMR-NOESY experiments [122]. Although the results of Yamashina et al. are remarkable, providing a defined criterion design for new sucrose receptors with high selectivity, the lack of signal transduction for temple receptors still limits its use for sucrose quantification in real samples.

#### 4.2.2. Aryl Boronic Acids

**Figure 12:**
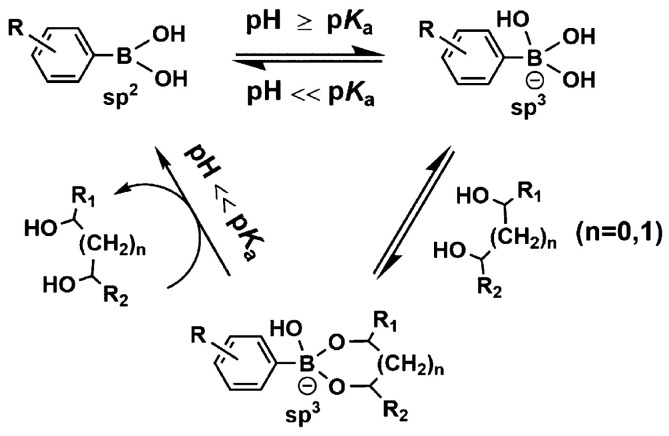
Reaction scheme for the complex formation of aryl boronic acids with diols. Figure taken from the reference [120].

Aryl boronic acids have been widely used in the design of ligands for the selective quantification of carbohydrates, especially monosaccharides. Aryl boronic acids bind covalently and reversibly with vicinal hydroxyl groups of carbohydrates at 1, 2, or 1, 3 positions, to form a borodiester ring of five or six atoms, respectively, [94,118,120]. Figure 12 shows the equilibrium species for the reversible reaction of carbohydrates with aryl boronic acids. The boron atom geometry changes from trigonal to tetragonal, with a negative formal charge, when the borodiester cycle is formed. This equilibrium is biased towards the tetragonal anionic product when the pH is greater than or equal to the p$K_{a}$ of aryl boronic acid [120,121].

**Figure 13:**
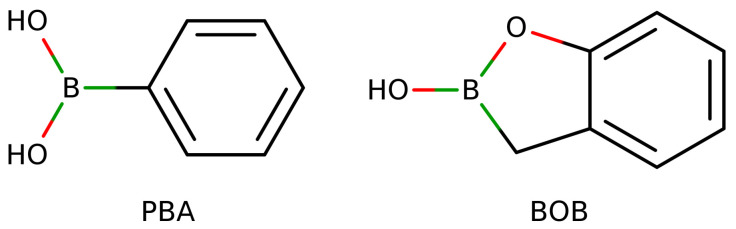
Phenyl Boronic Acid (PBA) and Benzoxaborole (BOB) structures.

Some researchers stated chemical and molecular structural criteria for ligand design of aryl boronic acids with high selectivity towards the desired carbohydrate. Yan et al. [131] reported the optimal pH for the binding of aryl boronic acids with carbohydrates, according to the equation:

(2) $${\mathrm{pH}}_{optimal}=\frac{pK_{a-acid}+pK_{a-diol}}{2},$$

where the $pK_{a-acid}$ and $pK_{a-diol}$ are the acidity constants for aryl boronic acid and the carbohydrate, respectively. Figure 13 shows the aryl boronic acids PBA and BOB, commonly used in the design of ligands for monosaccharides. The pKa of BOB is equal to 7.3, and the pKa of PBA is equal to 8.7. Nonetheless, the aromatic substitution with electroactive groups allows the pKa modulation of aryl boronic acids for optimal binding with carbohydrates according to required conditions [120]. This pKa modulation, and the reversible reaction with carbohydrates, make ligands based on aryl boronic acids optimal candidates to develop sensors for carbohydrate quantification under in vivo conditions.

**Figure 14:**
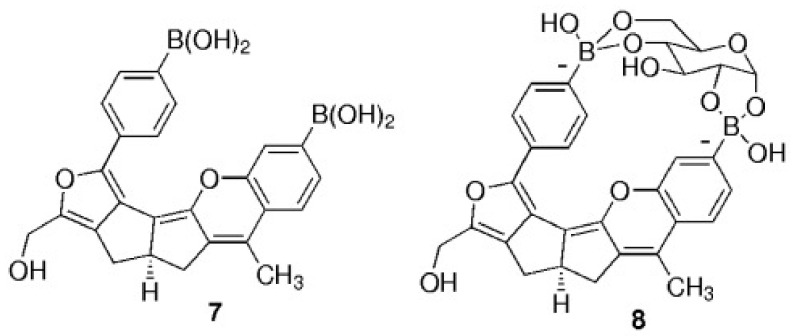
Computational aided designed ligand, based on PBA, for the selective binding to glucose. The molecule **7** correspond to the ligand in its free form, and the molecule **8** corresponds to the ligand-glucose complex. Figure taken from the reference [117].

The orientation and positioning of boronic groups are molecular structural criteria for the design of aryl boronic ligands. The selectivity of an aryl boronic ligand towards a carbohydrate and stability of the cyclic borodiester depend on steric effects [132], orientation, and relative position of hydroxyl groups, allowing boronic acid ligands to differentiate structurally similar saccharide molecules [94]. For example, one PBA molecule binds preferably to fructose over glucose with $K_{b}$ coefficients of 4370 M${}^{-1}$ and 110 M${}^{-1}$, respectively, [94]. On the other hand, glucose, unlike fructose, can bind two PBA molecules, simultaneously, at hydroxyl positions 1, 2 and 4, 6 [133,134]. Based on this feature, Yang et al. [117] designed a tweezer-like ligand with two PBA molecules to bind, preferably, glucose over fructose. Figure 14 shows the tweezer-like ligand designed by Yang et al. These authors realized a careful positioning of the PBA molecules (using computational chemistry calculations) to bind the hydroxyl groups of glucose at positions 1, 2 and 4, 6. A computer-aided design was performed to complete the tweezer-like ligand. In this way, Yang et al. designed a ligand (Figure 14) with a binding coefficient of $K_{b}=40,000\;M^{-1}$ for glucose, 400 times greater than $K_{b}$ for fructose [117] (A more comprehensive review about tweezer-like ligands based on PBA is found in reference [134]).

Double binding of PBA molecules to glucose [124,125,135,136] is the base for the design of sandwich-type sensors, which has achieved glucose quantification at molecular levels with high selectivity [124,125,135,136]. These sensors were made-up of surface and gold nanoparticles, functionalized with 4-mercaptophenylboronic acid (4-MPBA). Glucose reacts with 4-MPBA molecules assembled both on the gold surface and in the gold nanoparticle. The proximity of the nanoparticle with the gold surface allows glucose quantification either by the Raman spectrum amplified by the surface plasmon [125,135], or the change in the refractive index of the gold surface, due to the proximity of the gold nanoparticles attached to glucose by the 4-MPBA [124,136]. The change of the refractive index of the gold surface has allowed glucose quantification at pM-nM concentrations [124].

Yang et al. [128] reported a hybrid receptor composed of a PBA tweezer-like ligand [123,137] and aptamers, to tune the selectivity of the receptor towards glucose, fructose, or galactose. The hybrid receptor is based on the synergy between the PBA ligand and the aptamer; the PBA ligand provides the binding interaction towards the monosaccharide, and the aptamer provides a rigid framework and a tuned cavity for the PBA ligand-monosaccharide complex [128]. In this way, Yang et al. designed three different hybrid receptors with tuned selectivity towards glucose, fructose, or galactose with LOD of 1.7 μM, 2.0 μM, and 3.3 μM, respectively, [128]. Nevertheless, the development of new protocols has allowed the design of aptamers in the solution phase with the simultaneous presence of the target analyte and the interferents [129]. In this way, Nakatsuka et al. developed an aptamer that interacts directly with glucose and changes its conformation when bonded to it [129], allowing its quantification at pM and nM concentrations, under physiological conditions, when this aptamer was coupled to FET [129]. Recently, the aptamer developed by Nakatsuka et al., modified with a FRET reporter and bonded to another tiol-functionalized aptamer, has been successfully delivered into cell plants for glucose quantification [138]. The nanoscopic dimension of the aptamers (less than 20 nm) allowed the permeation of the cell wall, and the tiol functionalization allowed the transport of the aptamers through the cell membrane, into the cytoplasm, by the cell tiol-mediated uptake [138].

Despite the extensive research on aryl boronic acids for fructose and glucose detection, reports of aryl boronic acids for sucrose quantification are scarce [126,139,140]. Usually, sucrose is not the principal analyte for detection with aryl boronic ligands. Instead, sucrose quantification is performed as part of the screening process to assess the ligand selectivity among a series of carbohydrates. Nevertheless, it is worth noting the report by Zhang et al. [140], in which they synthesized a PBA ligand with a double binding site that, despite being selective for lactose ($K_{b}=35\;M^{-1}$), made it possible to quantify the disaccharides of a mixture, including sucrose, by means of a Linear Decomposition Analysis (LDA) of the fluorescence spectrum, achieving the sucrose quantification in the mM concentrations.

We want to remark on the versatility of ligands based on aryl boronic to be integrated into a wide variety of sensing platforms to quantify carbohydrates, which the following stand out: fluorescence quenching or enhancement [139,140,141,142,143], FRET [144,145], voltammetric [142,146] and potentiometric devices [147], Field Effect Transistors (FET) [126,129] and Surface Enhanced Raman Spectroscopy (SERS) [124,135,136,148]. This versatility is another appealing characteristic of aryl boronic acids to develop portable instruments for sucrose quantification on the field, in real-time wide, avoiding the use of expensive instruments and highly trained personnel to carry out the measurements.

### 4.3. Molecularly Imprinted Polymers-Based Sensors, as Chemochemical Cavity Receptors

**Figure 15:**
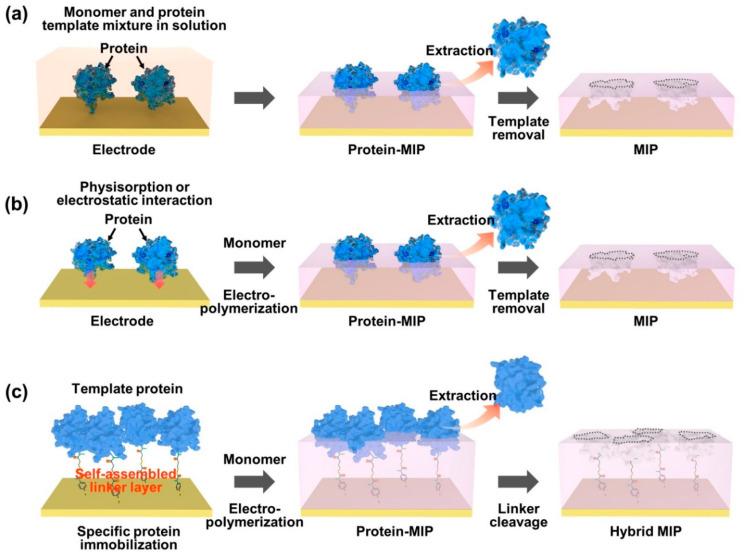
Representative strategies on the polymer surface imprinting process to construct specific recognition cavities (shown here for proteins, but equally applicable to smaller molecules). In an appropriate design concept, the selective rebinding site can be generated by using a functional monomer for electropolymerization on a prepared electrode surface, which includes the formation of the pre-polymerization complex (**a**), the template physisorption (**b**) and the immobilization of the target protein (**c**). Figure from reference [162].

A biomimetic design strategy has also been used to selectively bind and recognize sugars, including sucrose. Molecularly imprinted polymers (MIPs) [149], consisting of crosslinked polymer matrices with molecular recognition sites synthesized in the presence of a complementary target template fall into this category [150]. MIPs can be considered synthetic chemocavities, as tailor-made artificial receptors for sugars [80,151,152,153,154], metabolites [155,156], proteins [157,158], and other biomarkers [159] that recognize and bind analytes with high selectivity and chemical affinity. The MIP matrices can be synthesized simply by polymerization of monomers forming a complex with target molecules, in which a relatively weak bonding is set between the template molecules and crosslinked monomers. The fabrication process involves starting with the prepolymer/template mixture, in which the spatial arrangement of MIPs is determined by hydrogen bonds [160], Van der Waals forces, hydrophobic and/or electrostatic interactions [161]. The template molecules are then removed from the crosslinked polymeric matrices, which leaves cavities with specific chemical end groups that act as complementary templates defined by size, shape and chemical functionality. These can be made to be highly targetable recognition systems for specific molecules, such as chemical sensors, analytic separation membranes, solid-phase extractions, drug delivery systems, catalysts and library screening methods. Figure 15 provides a graphical summary of the MIP fabrication process.

Kajisa and Sakata [150] have designed a FET device consisting of a gate terminal coated with an MPI gel for selective binding to glucose. This MPI gel, based on PBA, is 200 times more selective for glucose than fructose, and charges negatively after binding with glucose, modifying the capacitance of the FET sensor. In this way, Kajisa and Sakata [150] achieved glucose quantification with LOD of 3.0 μM, and detection ranges from 100 μM to 4.0 mM. Shekarchizadeh et al. [80] reported a MIP system made of multiwall carbon nanotubes, a glassy carbon electrode, *o*-phenylenediamine as crosslinked polymer, and sucrose as a molecular template, with a performance similar to achieved for glucose. Shekarchizadeh et al. [80] reported a detection range of 0.01 mM–10.0 mM with a LOD of 3 μM for quantification of sucrose in sugar beet juices.

## 5. Conclusions and Perspectives

**Figure 16:**
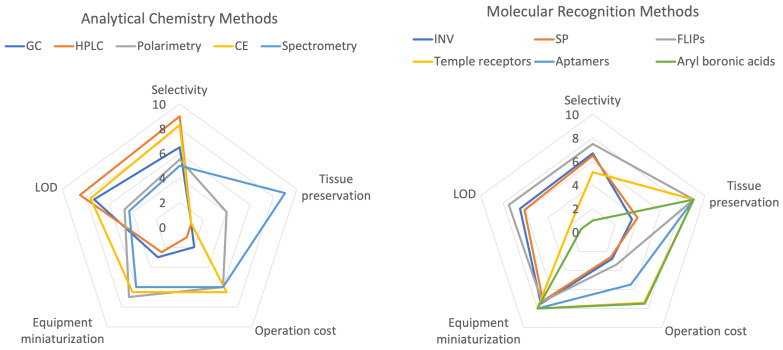
Qualitative comparison, on a scale from 0 (worse) to 10 (best), between the analytical chemistry (**left**) and the molecular recognition methods (**right**) for the quantification and detection of sucrose. We selected the LOD and selectivity for sucrose, tissue preservation, operation cost, and the miniaturization of the equipment, as important criteria to evaluate the potential use of these techniques for real-time sucrose quantification under in vivo conditions.

In Figure 16, we provide a graphical qualitative comparison of the main features of the techniques reviewed in this work for real-time quantification of sucrose at ultra-low concentrations, with application to plant breeding, epigenomic characterization of plants, and plant condition monitoring. High operation costs, robust equipment, and large facilities limit the use of HPLC and GC for the quantification of sucrose under lab conditions.

Polarimetry, although easy to use, may be replaced by CE, in the near future, as the method for routine quantification of sucrose on the field. The capability to identify other analytes than sucrose in the same run, simple instrumentation, and high potential for the design of hand-size instruments, are some of the reasons for replacing polarimetry with CE. On the other hand, tissue destruction by sample preparation forbids the use of CE for in vivo analysis of sucrose.

Spectroscopy is an analytical technique that enables the quantification of sucrose under in vivo conditions. The absence of sample pretreatment allows the preservation of the sample and tissue. However, the poor LODs of spectroscopy methods limit their use for sucrose quantification at ultralow concentrations. Machine learning techniques can complement the chemometric protocol for sucrose quantification with spectroscopy methods, improving their LODs.

Quantification of sucrose by means of biosensors-based INV, SP, and FLIP proteins has achieved a LOD at the μM level. Problems related to the selectivity, low binding constants $K_{b}$, and signal transduction for the protein-sucrose binding event, are the main challenges to improving the LOD for sucrose detection. Simultaneous hydrogen bonds with the -OH groups in carbon atoms C2, C3, C3’, and C’4 may be key to improving the selectivity, and $K_{b}$ for protein-sucrose binding. The hydrogen bonds just mentioned are not present in any of the CBS for the proteins covered in this review. Correct positioning of glutamate and aspartate residues in CBS, to bind the -OH in 1,2 positions of sucrose, may be one of the targets in protein design for sucrose biosensors to improve the LODs.

Natural lectins and temple receptors attract carbohydrates by non-covalent interactions and lack catalytic activity, therefore present similar limitations for their use in sucrose quantification, such as low $K_{b}$ binding coefficients, selectivity, and lack of signal transduction for the sucrose-binding event. The results obtained for the selective binding of sucrose nanocapsules based on anthracene and Pd(II) [122], offer another design route for highly selective ligands for sucrose. A spherical cavity with a dimensions diameter of 1 nm, and a volume > 300 Å${}^{3}$ allows ∼100% selectivity to sucrose, where the stacking interactions of the inner faces of the cavity with H atoms of sucrose are those responsible for the link [122]. These interactions could be mimicked in CBS proteins, e.g., by the interaction between the tryptophan residue with the hydrophobic faces of glucosyl and fructosyl moieties in sucrose. Lectins coupled to nanomaterials like carbon nanotubes, graphene, or metallic nanoparticles could be used to improve the signal transduction for the protein-sucrose binding.

Aryl boronic acids have shown ample versatility for signal transduction with carbohydrates, allowing a LOD in the pM-nM range glucose detection. Nevertheless, quantifying sucrose (and disaccharides in general) with aryl boronic acids has not achieved such performance.

Molecular recognition methods such as FLIPs, and synthetic receptors, are expected to gather most of the characteristics for in vivo quantification of sucrose, such as biocompatibility and no sample treatment, tissue preservation, low operation cost, and simple instrumentation. The hydrolysis reaction of sucrose by INV and SP enzymes, and the potential modification of the central metabolism of the plant, prescribe the use of INV and SP biosensors to ex situ measures of sucrose. Additionally, known problems and the high cost of enzyme immobilization increase the operation cost for INV and SP biosensors.

However, the development of natural or synthetic receptors with high selectivity for sucrose may be improved with the use of geometrical design criteria for tailor-made ligands. Small conformational deviations between solvated and crystalline sucrose, validated the use of sucrose geometry from crystalline data as a template for ligand design; a rigid and spherical cavity with 10 nm of diameter and volume > 300 Å${}^{3}$ to host sucrose by C-H ⋯π interactions; optimal positioning and orientation of carboxylate groups to form hydrogen bonds with the hydroxyls in carbon atoms C2, C3, C3’, and C’4 of sucrose; and the optimal positioning and orientation of PBA ligands for covalent bonds with the hydroxyls in 1, 3 positions, are some design criteria that may improve the selectivity of tailor-made ligands for sucrose. Moreover, these design criteria can be easy coded for the computer-aided design of proteins, temple receptors, aptamers, and tweezer-like ligands based on PBA, or MIPs, among others.

In addition to selectivity, delivery into desired tissue or cellular compartments, and the transduction process for a high signal-to-noise ratio, are common overt challenges to natural and synthetic receptors for sucrose quantification. Although aptamers have been successfully delivered into the cytosol of animal and plant cells, and FET-coupled to aptamers and sandwich-type sensors for glucose has achieved remarkable LODs on the order of pM-nM; delivery of ligands and acquisition of signals other than light-matter interaction, under in vivo conditions, remains as a difficult task.

## Figures and Tables

**Table 1:** Some of the physical properties of sucrose. Table adapted from [27].

Property	Value
Melting point (${}^{\circ }$C)	160–186
Specific rotation (${\left[\alpha \right]}_{D}^{20}$ in deg)	+66.53
Solubility in water at 20 ${}^{\circ }$C (g/mL)	2.00
Heat of solution (kJ/mol)	4.75 ± 0.26
Dipole moment (C m)	3.1 × 10${}^{-18}$
Normal entropy (J mol${}^{-1}$ K${}^{-1}$)	360.5

**Table 2:** Analytical methods for sucrose quantification.

Technique	Sample	LOD	LOQ	Detection Range	Reference
**Polarimetry**					
Polarimetry	Iron sucrose raw material	0.222 mM	0.674 mM	29.2 mM–204.5 mM	[32]
Polarimetry	Sucrose hydrolysis by INV in solution			2.28 μM–50.2 mM	[29]
**Liquid chromatography**					
HPLC-ELSD	Fruits	0.788 mM	2.658 mM		[33]
HPLC-(C-CAD)-ELSD	Food samples	33.0 μM		73.0 μM–5.8 mM	[34]
HPLC-CAD	Fruit beverages and dietary supplements	0.35 μM	1.16 μM	2.92 μM–292.14 μM	[35]
HPLC-RI	Milk based formulae	0.46 mM	0.75 mM	1.4 mM–29.1 mM	[36]
HPLC-RI	Food samples	0.29 mM			[37]
HPLC-PAD	Fruits and vegeteables	0.861 μM	2.86 μM		[38]
HPLC-PAD	Honeydew and nectar	22.4 μM	74.7 μM	29.2 μM–58.4 mM	[39]
HPLC-MS	Human urine	50.0 nM	0.149 μM		[40]
HPLC-PAD	Biomass	3.5 nM	11.3 nM	64.2 nM–502.4 nM	[41]
HPLC-PAD	Raw sugar	1.5 μM	3.0 μM	3.0 μM–3.0 μM	[42]
HPLC-RI	Leaf and fruit peel apple	9.37 μM	31.3 μM	0.809 mM–16.1 mM	[43]
HPLC-MS	Intracellular extracts of cyanobacteria	58.4 nM	0.20 μM	0.14 μM–29.2 μM	[44]
UHPLC-HRMS-ESI	in vitro laboratory solution	0.24 μM	0.84 μM		[45]
UHPLC-MS-ESI	Palm friut	0.73 μM	3.09 μM	2.9 μM–146.1 μM	[46]
Size exclusion chromatography and ELSD	Lyophilized thrombin powder	0.292 mM	0.043 mM		[47]
LC-MS-ESI	Radix Rehmanniae root	7.0 nM	19.7 nM	4.43 nM–886.3 nM	[48]
HPLC-ELSD	Carbonate cola drinks and fruit juices	0.262 mM		0.292 mM–2.92 mM	[49]
HPLC-ELSD	Jujube extract	6.7 μM	23.3 μM	29.2 μM–2.9 mM	[50]
**Gas chromatography**					
GC-MS	Aerosol particle, soil and sediment samples		0.818 μM	3.5 μM–350 μM	[51]
GC-FID	Tobacco samples	0.26 μM		8.76 μM–876.4 μM	[52]
GC-MS	Jujube extract	0.84 μM	2.8 μM	3.0 μM–3.0 mM	[50]
**Capilar electrophoresis**					
CE-DAD	Biomass			0.20 mM–7.0 mM	[53]
CE-DAD	Honey samples	64.2 μM	0.2 mM	0.5 mM–3.4 mM	[54]
CE-DAD	Forensic, pharmaceutical, and beverage samples	5.0 μM	15.0 μM	15.0 μM - 700.0 μM	[55]
CE-DAD	Wort samples	97.9 μM	0.3 mM	97.9 μM–1.2 mM	[56]
CE-CL		18.0 μM		50.0 μM–10.0 mM	[57]
CE-Conductometry	High-energy drinks	43.8 μM	0.15 mM		[58]
CE-Amperometry	Honey, milk	0.24 μM			[59]
CE-Amperometry	Honey, milk, peach, banana	0.42 μM			[60]
CE-Amperometry	Honey	0.6 μM		2.0 μM–1.0 mM	[61]
CE-Amperometry	Moutan cortex	1.2 μM		5.0 μM–2.0 mM	[62]
**Spectroscopy**					
Raman	Commercial soft drinks			2.92 M–43.82 M	[63]
Raman	Lemon-type soft drinks	5.84 mM	24.0 mM	24.0 mM–350.0 mM	[64]
IR	Commercial soft drinks			0.10 M–1.20 M	[65]
FT-NIR	Fruit juices			58.42 mM–233.0 mM	[66]
DOSY-qNMR	Fruit juices and sports drink			1.46 mM–58.42 mM	[67]
**Natural receptors**					
INV-FDH for amperometric detection	Condensed milk and infant food	0.36 μM		1.2 μM–3.0 mM	[68]
INV-Mut/PO${}_{4}^{-3}$-GOx and amperometric detection	Fruit juices			0.1 mM–2.5 mM	[69]
INV-Mut/PO${}_{4}^{-3}$-(FAD-GDH) biosensor and amperometric detection	Green coffe samples	8.4 μM		10 μM–1200 μM	[70]
μPAD-INV-GOx and Colorimetric detection	Sugar juice plants	0.90 mM		1.0 mM–25 mM	[71]
INV-GOx and Fluorimetric detection	Root beet extracts			1 μM–16 μM	[72]
INV-HK-G6PDH-PGI and spectrophotometric detection		20.0 μM		80.0 μM–12.0 mM	[73]
INV-GOx-CAT thermistor biosensor				0.1 mM–50.0 mM	[74]
SP-PGM-G6PDH and amperometric detection	Fruit juices	1.0 mM		1.0 mM–15.0 mM	[75]
SP-PGM-G6PDH and and Fluorimetric detection	Soft drinks	0.1 μM		0.1 μM–200.0 μM	[76]
Genetically encoded FRET sensor FLIPsuc-90μΔ1 mutants	Pure solutions of sucrose			7 μM–585 μM	[14]
**Miscellaneous**					
**AgNPs-colorimetric detection**	Food samples			0.1 mM–1.0 mM	[77]
RF(THz)	Aqueous sucrose solution			0.438–2.921 ± 0.146 M	[78]
Lossy Mode Resonance-Based Refractive Index				0.6 M–1.8 M	[79]
Molecular Imprinted Polymers	Sucrose in sugar beet juices	3.0 mM		0.01mM–10.0 mM	[80]

LOD: Limit of detection. LOQ: Limit of quantification. RF: Radio frequency.

**Table 3:** Vibrational frequencies used for the sucrose quantification with PLS analysis [63].

Band (cm${}^{-1}$)	Band Assigment
824	CH out-of-plane deformation
911	CH${}_{2}$ out-of-plane wagging
1052	C–O stretching
1119	C–O stretching
1255	C–O–C antisymmetric stretching
1445	CH${}_{2}$ scissoring vibration

**Table 4:** Specific activity (in U/mg units) of *S*INV. Table from reference [97].

Substrate	Activity
Sucrose	520 ± 20 ${}^{a}$
1-ketose	102 ± 11 ${}^{a}$
Nystose	36 ± 1 ${}^{a}$
Raffinose	187 ± 8 ${}^{a}$
Inulin	2.5 ± 0.1 ${}^{b}$

*^a^* The substrate concentration was 250 mM. *^b^* The substrate concentration was 10%.

**Table 5:** Monosaccharides and disaccharides detected with natural or artificial receptors.

Receptor	Analyte	$K_{d}$	LOD	Detection Range	Detector	References
**Natural receptors—FLIPs and Lectins**						
FLIPsuc-90μΔ1	Sucrose	65.0 μM		7 μM–585 μM	Fluorescence	[14]
FLIPsuc-4μΔ1	Sucrose	3.7 μM			Fluorescence	[90]
FLIPsuc-4μΔ1 W283A	Sucrose	88.0 μM			Fluorescence	[90]
Lectins	Monosaccharides	2.0 mM–10.0 mM				[94,95]
**Synthetic receptors—Temple molecules**						
Temple receptor	Glucose	54.9 μM			NMR, ITC	[95]
Temple receptor	β-D-Cellobiose	1.0 mM			NMR, ITC, CD, Fluorescence	[114]
Temple receptor	D-Cellobiose	1.7 mM			NMR, ITC, CD, Fluorescence	[114]
M${}_{2}$L${}_{4}$ Nanocapsule	Sucrose	909.0 μM			NMR-NOESY	[122]
**Synthetic receptors—Aryl Boronic Acids**						
PBA	Fructose	228.8 μM			Fluorescence	[94]
PBA	Glucose	9.0 mM			Fluorescence	[94]
Computational designed tweezer ligand	Fructose	10.0 mM			Fluorescence	[117]
Computational designed tweezer ligand	Glucose	25.0 μM			Fluorescence	[117]
Shinkai ligand	Glucose	3.8 mM			Fluorescence	[123]
Sandwich type sensor	Glucose		295.0 pM	1.0 nM–1.0 μM	Plasmonic fiber sensor	[124]
Sandwich type sensor	Glucose		10 nM	10.0 nM–10.0 mM	SERS	[125]
MPBA	Sucrose		7.9 μM	50 μM–40 mM	FET	[126]
PBA-SWCNT	Sucrose		2.5 mM	1 mM–30 mM	Chemresistor	[127]
**Aptamers**						
Shinkai ligand + aptamer	Glucose	1.7 μM		1.0 μM–10.0 μM	Fluosrescence	[128]
Aptamer	Glucose	10.0 mM		10.0 pM–10.0 nM	FET	[129]

LOD: Limit of detection. ITC: Isothermic Titration Calorimetry. CD: Circular Dichroism. *K* = 1/*K_b_*.

## Data Availability

Not applicable.

## References

[1] Porter J.R., Xie L., Challinor A.J., Cochrane K., Howden S.M., Iqbal M.M., Lobell D.B., Travasso M.I., Field C.B., Barros V.R., Dokken D.J., Mach K.J., Mastrandrea M.D., Bilir T.E., Chatterjee M., Ebi K.L., Estrada Y.O., Genova R.C. (2014). 2014: Food security and food production systems. Climate Change 2014: Impacts, Adaptation, and Vulnerability. Part A: Global and Sectoral Aspects. Contribution of Working Group II to the Fifth Assessment Report of the Intergovernmental Panel on Climate Change.

[2] Cline W.R. (2008). Global Warming and Agriculture. Financ. Dev..

[3] Pounds J.A., Puschendorf R. (2004). Clouded futures. Nature.

[4] Chakraborty S., Newton A.C. (2011). Climate change, plant diseases and food security: An overview. Plant Pathol..

[5] Rengel Z., Singh B.P., Cowie A.L., Chan K.Y. (2011). Soil pH, Soil Health and Climate Change. Soil Health and Climate Change.

[6] Razzaq A., Kaur P., Akhter N., Wani S.H., Saleem F. (2021). Next-Generation Breeding Strategies for Climate-Ready Crops. Front. Plant Sci..

[7] Ceccarelli S., Grando S., Maatougui M., Michael M., Slash M., Haghparast R., Rahmanian M., Taheri A., Al-Yassin A., Benbelkacem A. (2010). Plant breeding and climate changes. J. Agric. Sci..

[8] Dreccer M.F., Bonnett D., Lafarge T., Christou P., Savin R., Costa-Pierce B.A., Misztal I., Whitelaw C.B.A. (2013). Plant Breeding Under a Changing Climate. Sustainable Food Production.

[9] Varotto S., Krugman T., Aiese Cigliano R., Kashkush K., Kondić-Špika A., Aravanopoulos F.A., Pradillo M., Consiglio F., Aversano R., Pecinka A. (2022). Exploitation of epigenetic variation of crop wild relatives for crop improvement and agrobiodiversity preservation. Theor. Appl. Genet..

[10] Halford N., Curtis T., Muttucumaru N., Postles J., Mottram D. (2011). Sugars in crop plants. Ann. Appl. Biol..

[11] Yoon J., Cho L.H., Tun W., Jeon J.S., An G. (2021). Sucrose signaling in higher plants. Plant Sci..

[12] Ciereszko I. (2018). Regulatory roles of sugars in plant growth and development. Acta Soc. Bot. Pol..

[13] Okumoto S., Jones A., Frommer W.B. (2012). Quantitative Imaging with Fluorescent Biosensors. Annu. Rev. Plant Biol..

[14] Sadoine M., Reger M., Wong K.M., Frommer W.B. (2021). Affinity Series of Genetically Encoded Förster Resonance Energy-Transfer Sensors for Sucrose. ACS Sens..

[15] Deuschle K., Chaudhuri B., Okumoto S., Lager I., Lalonde S., Frommer W.B. (2006). Rapid Metabolism of Glucose Detected with FRET Glucose Nanosensors in Epidermal Cells and Intact Roots of Arabidopsis RNA-Silencing Mutants. Plant Cell.

[16] Chaudhuri B., Hörmann F., Lalonde S., Brady S.M., Orlando D.A., Benfey P., Frommer W.B. (2008). Protonophore- and pH-insensitive glucose and sucrose accumulation detected by FRET nanosensors in Arabidopsis root tips. Plant J..

[17] Chen L.Q., Qu X.Q., Hou B.H., Sosso D., Osorio S., Fernie A.R., Frommer W.B. (2012). Sucrose Efflux Mediated by SWEET Proteins as a Key Step for Phloem Transport. Science.

[18] Eom J.S., Luo D., Atienza-Grande G., Yang J., Ji C., Thi Luu V., Huguet-Tapia J.C., Char S.N., Liu B., Nguyen H. (2019). Diagnostic kit for rice blight resistance. Nat. Biotechnol..

[19] Sosso D., Luo D., Li Q.B., Sasse J., Yang J., Gendrot G., Suzuki M., Koch K.E., McCarty D.R., Chourey P.S. (2015). Seed filling in domesticated maize and rice depends on SWEET-mediated hexose transport. Nat. Genet..

[20] Li J., Liesche J. (2022). Strategies for Measuring Cytosolic Sugar Concentrations in Plant Cells. Annual Plant Reviews Online.

[21] Lowman D.W. (1978). Bibliography: Methods of sucrose analysis. J. Sugar Beat Res..

[22] Pokrzywnicka M., Koncki R. (2018). Disaccharides Determination: A Review of Analytical Methods. Crit. Rev. Anal. Chem..

[23] Brown G.M., Levy H.A. (1973). Further refinement of the structure of sucrose based on neutron-diffraction data. Acta Crystallogr. Sect. B.

[24] Rozada T.D.C., Pontes R.M., Rittner R., Basso E.A. (2016). Stereoelectronic effects of the glycosidic linkage on the conformational preference of d-sucrose. RSC Adv..

[25] Xia J., Case D.A. (2012). Sucrose in aqueous solution revisited, Part 1: Molecular dynamics simulations and direct and indirect dipolar coupling analysis. Biopolymers.

[26] Feng S., Bagia C., Mpourmpakis G. (2013). Determination of Proton Affinities and Acidity Constants of Sugars. J. Phys. Chem. A.

[27] Godshall M.A., Eggleston G., Thompson J., Kochergin V.S. (2021). Kirk-Othmer Encyclopedia of Chemical Technology.

[28] O’Neil M.J. (2001). The Merck Index: An Encyclopedia of Chemicals, Drugs, and Biologicals.

[29] Li D., Weng C., Ruan Y., Li K., Cai G., Song C., Lin Q. (2021). An Optical Chiral Sensor Based on Weak Measurement for the Real-Time Monitoring of Sucrose Hydrolysis. Sensors.

[30] Wang R., Zhou J., Zeng K., Chen S., Ling X., Shu W., Luo H., Wen S. (2020). Ultrasensitive and real-time detection of chemical reaction rate based on the photonic spin Hall effect. APL Photonics.

[31] Skoog D.A., West D.M., Holler F.J., Crouch S.R. (2013). Fundamentals of Analytical Chemistry.

[32] Desai H., Sevak M., Panchal V., Panchal K., Patel N. (2013). A new Polarimeteric method for the analysis of Sucrose in iron sucrose raw material, Iron Sucrose Injection and Inprocess Bulk Formulations. Int. J. Pharm. Sci. Res..

[33] Ma C., Sun Z., Chen C., Zhang L., Zhu S. (2014). Simultaneous separation and determination of fructose, sorbitol, glucose and sucrose in fruits by HPLC–ELSD. Food Chem..

[34] Márquez-Sillero I., Cárdenas S., Valcárcel M. (2013). Comparison of two evaporative universal detectors for the determination of sugars in food samples by liquid chromatography. Microchem. J..

[35] Grembecka M., Lebiedzińska A., Szefer P. (2014). Simultaneous separation and determination of erythritol, xylitol, sorbitol, mannitol, maltitol, fructose, glucose, sucrose and maltose in food products by high performance liquid chromatography coupled to charged aerosol detector. Microchem. J..

[36] Chávez-Servín J.L., Castellote A.I., López-Sabater M. (2004). Analysis of mono- and disaccharides in milk-based formulae by high-performance liquid chromatography with refractive index detection. J. Chromatogr. A.

[37] Zakharova A.M., Grinshtein I.L., Kartsova L.A. (2013). Determination of carbohydrates and sweeteners in foods and biologically active additives by high-performance liquid chromatography. J. Anal. Chem..

[38] Wang B., Wang X., Bei J., Xu L., Zhang X., Xu Z. (2021). Development and Validation of an Analytical Method for the Quantification of Arabinose, Galactose, Glucose, Sucrose, Fructose, and Maltose in Fruits, Vegetables, and Their Products. Food Anal. Methods.

[39] Ni C., Zhu B., Wang N., Wang M., Chen S., Zhang J., Zhu Y. (2016). Simple column-switching ion chromatography method for determining eight monosaccharides and oligosaccharides in honeydew and nectar. Food Chem..

[40] Kubica P., Kot-Wasik A., Wasik A., Namieśnik J., Landowski P. (2012). Modern approach for determination of lactulose, mannitol and sucrose in human urine using HPLC–MS/MS for the studies of intestinal and upper digestive tract permeability. J. Chromatogr. B.

[41] Sevcik R.S., Mowery R.A., Becker C., Chambliss C.K. (2011). Rapid analysis of carbohydrates in aqueous extracts and hydrolysates of biomass using a carbonate-modified anion-exchange column. J. Chromatogr. A.

[42] Suksom W., Wannachai W., Boonchiangma S., Chanthai S., Srijaranai S. (2015). Ion Chromatographic Analysis of Monosaccharides and Disaccharides in Raw Sugar. Chromatographia.

[43] Filip M., Vlassa M., Coman V., Halmagyi A. (2016). Simultaneous determination of glucose, fructose, sucrose and sorbitol in the leaf and fruit peel of different apple cultivars by the HPLC–RI optimized method. Food Chem..

[44] Fa Y., Liang W., Cui H., Duan Y., Yang M., Gao J., Liu H. (2015). Capillary ion chromatography–mass spectrometry for simultaneous determination of glucosylglycerol and sucrose in intracellular extracts of cyanobacteria. J. Chromatogr. B.

[45] Barzen-Hanson K.A., Wilkes R.A., Aristilde L. (2018). Quantitation of carbohydrate monomers and dimers by liquid chromatography coupled with high-resolution mass spectrometry. Carbohydr. Res..

[46] Ghfar A.A., Wabaidur S.M., Ahmed A.Y.B.H., Alothman Z.A., Khan M.R., Al-Shaalan N.H. (2015). Simultaneous determination of monosaccharides and oligosaccharides in dates using liquid chromatography–electrospray ionization mass spectrometry. Food Chem..

[47] Li F., Zhang H., Li Y., Yu Y., Chen Y., Xie M., Duan G. (2012). Simultaneous Identification and Quantification of Dextran 20 and Sucrose in Lyophilized Thrombin Powder by Size Exclusion Chromatography with ELSD. Chromatographia.

[48] Liu Z., Lou Z., Ding X., Li X., Qi Y., Zhu Z., Chai Y. (2013). Global characterization of neutral saccharides in crude and processed Radix Rehmanniae by hydrophilic interaction liquid chromatography tandem electrospray ionization time-of-flight mass spectrometry. Food Chem..

[49] Li J., Chen M., Zhu Y. (2007). Separation and determination of carbohydrates in drinks by ion chromatography with a self-regenerating suppressor and an evaporative light-scattering detector. J. Chromatogr. A.

[50] Sun S., Wang H., Xie J., Su Y. (2016). Simultaneous determination of rhamnose, xylitol, arabitol, fructose, glucose, inositol, sucrose, maltose in jujube (Zizyphus jujube Mill.) extract: Comparison of HPLC-ELSD, LC-ESI-MS/MS and GC-MS. Chem. Cent. J..

[51] Medeiros P.M., Simoneit B.R. (2007). Analysis of sugars in environmental samples by gas chromatography—Mass spectrometry. J. Chromatogr. A.

[52] Cai K., Hu D., Lei B., Zhao H., Pan W., Song B. (2015). Determination of carbohydrates in tobacco by pressurized liquid extraction combined with a novel ultrasound-assisted dispersive liquid–liquid microextraction method. Anal. Chim. Acta.

[53] Aid T., Paist L., Lopp M., Kaljurand M., Vaher M. (2016). An optimized capillary electrophoresis method for the simultaneous analysis of biomass degradation products in ionic liquid containing samples. J. Chromatogr. A.

[54] Rizelio V.M., Tenfen L., da Silveira R., Gonzaga L.V., Costa A.C.O., Fett R. (2012). Development of a fast capillary electrophoresis method for determination of carbohydrates in honey samples. Talanta.

[55] Sarazin C., Delaunay N., Costanza C., Eudes V., Gareil P. (2012). Application of a new capillary electrophoretic method for the determination of carbohydrates in forensic, pharmaceutical, and beverage samples. Talanta.

[56] Cortacero Ramírez S., Segura Carretero A., Cruces Blanco C., de Castro M.H.B., Fernández Gutiérrez A. (2005). Indirect determination of carbohydrates in wort samples and dietetic products by capillary electrophoresis. J. Sci. Food Agric..

[57] Zhu J., Shu L., Wu M., Wang Z., Wang Q., He P., Fang Y. (2012). Development of a compact chemiluminescence system coupled with capillary electrophoresis for carbohydrate analysis. Talanta.

[58] Vochyánová B., Opekar F., Tůma P., Štulík K. (2012). Rapid determinations of saccharides in high-energy drinks by short-capillary electrophoresis with contactless conductivity detection. Anal. Bioanal. Chem..

[59] Liang P., Sun M., He P., Zhang L., Chen G. (2016). Determination of carbohydrates in honey and milk by capillary electrophoresis in combination with graphene—Cobalt microsphere hybrid paste electrodes. Food Chem..

[60] Chen Q., Zhang L., Chen G. (2012). Facile Preparation of Graphene-Copper Nanoparticle Composite by in Situ Chemical Reduction for Electrochemical Sensing of Carbohydrates. Anal. Chem..

[61] Cheng X., Zhang S., Zhang H., Wang Q., He P., Fang Y. (2008). Determination of carbohydrates by capillary zone electrophoresis with amperometric detection at a nano-nickel oxide modified carbon paste electrode. Food Chem..

[62] Chen G., Zhang L., Zhu Y. (2006). Determination of glycosides and sugars in Moutan Cortex by capillary electrophoresis with electrochemical detection. J. Pharm. Biomed. Anal..

[63] Silveira L., Moreira L.M., Conceição V.G.B., Casalechi H.L., Muñoz I.S., Da Silva F.F., Silva M.A.S.R., De Souza R.A., Pacheco M.T.T. (2009). Determination of sucrose concentration in lemon-type soft drinks by dispersive Raman spectroscopy. Spectroscopy.

[64] Ilaslan K., Boyaci I.H., Topcu A. (2015). Rapid analysis of glucose, fructose and sucrose contents of commercial soft drinks using Raman spectroscopy. Food Control.

[65] Kemsley E., Zhuo L., Hammouri M., Wilson R. (1992). Quantitative analysis of sugar solutions using infrared spectroscopy. Food Chem..

[66] Rodriguez-Saona L.E., Fry F.S., McLaughlin M.A., Calvey E.M. (2001). Rapid analysis of sugars in fruit juices by FT-NIR spectroscopy. Carbohydr. Res..

[67] Cao R., Nonaka A., Komura F., Matsui T. (2015). Application of diffusion ordered-1H-nuclear magnetic resonance spectroscopy to quantify sucrose in beverages. Food Chem..

[68] Vargas E., Gamella M., Campuzano S., Guzmán-Vázquez de Prada A., Ruiz M., Reviejo A., Pingarrón J. (2013). Development of an integrated electrochemical biosensor for sucrose and its implementation in a continuous flow system for the simultaneous monitoring of sucrose, fructose and glucose. Talanta.

[69] Majer-Baranyi K., Adányi N., Váradi M. (2008). Investigation of a multienzyme based amperometric biosensor for determination of sucrose in fruit juices. Eur. Food Res. Technol..

[70] Stredansky M., Redivo L., Magdolen P., Stredansky A., Navarini L. (2018). Rapid sucrose monitoring in green coffee samples using multienzymatic biosensor. Food Chem..

[71] Aksorn J., Teepoo S. (2020). Development of the simultaneous colorimetric enzymatic detection of sucrose, fructose and glucose using a microfluidic paper-based analytical device. Talanta.

[72] Trebbi D., McGrath J.M. (2004). Fluorometric Sucrose Evaluation for Sugar Beet. J. Agric. Food Chem..

[73] García de María C., Townsend A. (1992). Sequential determination of glucose, fructose and sucrose by flow-injection analysis with immobilized enzyme reactors and spectrophotometric detection. Anal. Chim. Acta.

[74] Mandenius C.F., Bülow L., Danielsson B., Mosbach K. (1985). Monitoring and control of enzymic sucrose hydrolysis using on-line biosensors. Appl. Microbiol. Biotechnol..

[75] Maestre E., Katakis I., Domínguez E. (2001). Amperometric flow-injection determination of sucrose with a mediated tri-enzyme electrode based on sucrose phosphorylase and electrocatalytic oxidation of NADH. Biosens. Bioelectron..

[76] Kogure M., Mori H., Ariki H., Kojima C., Yamamoto H. (1997). Determination of sucrose using sucrose phosphorylase in a flow-injection system. Anal. Chim. Acta.

[77] Della Pelle F., Scroccarello A., Scarano S., Compagnone D. (2019). Silver nanoparticles-based plasmonic assay for the determination of sugar content in food matrices. Anal. Chim. Acta.

[78] Aguila Rodriguez G., Arias Duque N.P., Gonzalez Sanchez B.E., Sandoval Gonzalez O.O., Giraldo Osorio O.H., Trujillo Romero C.J., Wilches Torres M.A., Flores Cuautle J.D.J.A. (2019). Sugar Concentration Measurement System Using Radiofrequency Sensor. Sensors.

[79] Saini R., Kumar A., Bhatt G., Kapoor A., Paliwal A., Tomar M., Gupta V. (2020). Lossy Mode Resonance-Based Refractive Index Sensor for Sucrose Concentration Measurement. IEEE Sens. J..

[80] Shekarchizadeh H., Ensafi A.A., Kadivar M. (2013). Selective determination of sucrose based on electropolymerized molecularly imprinted polymer modified multiwall carbon nanotubes/glassy carbon electrode. Mater. Sci. Eng. C.

[81] (2004). Mono- and Oligosaccharides. Handbook of Food Analytical Chemistry.

[82] Harvey D.J. (2011). Derivatization of carbohydrates for analysis by chromatography; electrophoresis and mass spectrometry. J. Chromatogr. B.

[83] de Almeida V.E., de Araújo Gomes A., de Sousa Fernandes D.D., Goicoechea H.C., Galvão R.K.H., Araújo M.C.U. (2018). Vis-NIR spectrometric determination of Brix and sucrose in sugar production samples using kernel partial least squares with interval selection based on the successive projections algorithm. Talanta.

[84] Xie L., Ye X., Liu D., Ying Y. (2009). Quantification of glucose, fructose and sucrose in bayberry juice by NIR and PLS. Food Chem..

[85] Huck C.W. (2015). Advances of infrared spectroscopy in natural product research. Phytochem. Lett..

[86] Pallua J.D., Pezzei C., Huck-Pezzei V., Schonbichler S.A., Bittner L.K., Bonn G.K., Saeed A., Majeed S., Farooq A., Najam-ul Haq M. (2011). Advances of Infrared Spectroscopic Imaging and Mapping Technologies of Plant Material. Curr. Bioact. Compd..

[87] Houhou R., Bocklitz T. (2021). Trends in artificial intelligence, machine learning, and chemometrics applied to chemical data. Anal. Sci. Adv..

[88] Guo S., Popp J., Bocklitz T. (2021). Chemometric analysis in Raman spectroscopy from experimental design to machine learning-based modeling. Nat. Protoc..

[89] Torrione P., Collins L., Morton K., Baudelet M. (2014). 5-Multivariate analysis, chemometrics, and machine learning in laser spectroscopy. Laser Spectroscopy for Sensing.

[90] Lager I., Looger L.L., Hilpert M., Lalonde S., Frommer W.B. (2006). Conversion of a Putative Agrobacterium Sugar-binding Protein into a FRET Sensor with High Selectivity for Sucrose. J. Biol. Chem..

[91] Sharma V., Surolia A. (1997). Analyses of carbohydrate recognition by legume lectins: Size of the combining site loops and their primary specificity. J. Mol. Biol..

[92] Casset F., Hamelryck T., Loris R., Brisson J.R., Tellier C., Dao-Thi M.H., Wyns L., Poortmans F., Pérez S., Imberty A. (1995). NMR, Molecular Modeling, and Crystallographic Studies of Lentil Lectin-Sucrose Interaction. J. Biol. Chem..

[93] Loris R., Imberty A., Beeckmans S., Van Driessche E., Read J.S., Bouckaert J., De Greve H., Buts L., Wyns L. (2003). Crystal Structure of Pterocarpus angolensis Lectin in Complex with Glucose, Sucrose, and Turanose. J. Biol. Chem..

[94] Wu X., Li Z., Chen X.X., Fossey J.S., James T.D., Jiang Y.B. (2013). Selective sensing of saccharides using simple boronic acids and their aggregates. Chem. Soc. Rev..

[95] Tromans R.A., Carter T.S., Chabanne L., Crump M.P., Li H., Matlock J.V., Orchard M.G., Davis A.P. (2019). A biomimetic receptor for glucose. Nat. Chem..

[96] Trollope K.M., van Wyk N., Kotjomela M.A., Volschenk H. (2015). Sequence and structure-based prediction of fructosyltransferase activity for functional subclassification of fungal GH32 enzymes. Febs J..

[97] Sainz-Polo M., Ramírez-Escudero M., Lafraya A., González B., Marín-Navarro J., Polaina J., Sanz-Aparicio J. (2013). Three-dimensional Structure of Saccharomyces Invertase: Role of a non-catalytic domain in oligomerization and substrate specificity. J. Biol. Chem..

[98] Lammens W., Le Roy K., Van Laere A., Rabijns A., Van den Ende W. (2008). Crystal Structures of Arabidopsis thaliana Cell-Wall Invertase Mutants in Complex with Sucrose. J. Mol. Biol..

[99] Mátrai J., Lammens W., Jonckheer A., Le Roy K., Rabijns A., Van den Ende W., De Maeyer M. (2008). An alternate sucrose binding mode in the E203Q Arabidopsis invertase mutant: An X-ray crystallography and docking study. Proteins Struct. Funct. Bioinform..

[100] Sturm A. (1999). Invertases. Primary Structures, Functions, and Roles in Plant Development and Sucrose Partitioning. Plant Physiol..

[101] Kulshrestha S., Tyagi P., Sindhi V., Yadavilli K.S. (2013). Invertase and its applications—A brief review. J. Pharm. Res..

[102] Goedl C., Sawangwan T., Wildberger P., Nidetzky B. (2010). Sucrose phosphorylase: A powerful transglucosylation catalyst for synthesis of *α*-D-glucosides as industrial fine chemicals. Biocatal. Biotransform..

[103] Sprogøe D., van den Broek L.A.M., Mirza O., Kastrup J.S., Voragen A.G.J., Gajhede M., Skov L.K. (2004). Crystal Structure of Sucrose Phosphorylase from Bifidobacterium adolescentis. Biochemistry.

[104] Franceus J., Desmet T. (2020). Sucrose Phosphorylase and Related Enzymes in Glycoside Hydrolase Family 13: Discovery, Application and Engineering. Int. J. Mol. Sci..

[105] Mirza O., Skov L.K., Sprogøe D., van den Broek L.A., Beldman G., Kastrup J.S., Gajhede M. (2006). Structural Rearrangements of Sucrose Phosphorylase from Bifidobacterium adolescentis during Sucrose Conversion. J. Biol. Chem..

[106] Singh R., Singh A., Sachan S., Kuddus M. (2019). Enzymes Used in the Food Industry: Friends or Foes?. Enzymes in Food Biotechnology.

[107] Bezrutczyk M., Hartwig T., Horschman M., Char S.N., Yang J., Yang B., Frommer W.B., Sosso D. (2018). Impaired phloem loading in zmsweet13a,b,c sucrose transporter triple knock-out mutants in Zea mays. New Phytol..

[108] Lin I.W., Sosso D., Chen L.Q., Gase K., Kim S.G., Kessler D., Klinkenberg P.M., Gorder M.K., Hou B.H., Qu X.Q. (2014). Nectar secretion requires sucrose phosphate synthases and the sugar transporter SWEET9. Nature.

[109] Sharon N., Lis H., Lennarz W.J., Lane M.D. (2013). Lectins. Encyclopedia of Biological Chemistry.

[110] Loris R., Hamelryck T., Bouckaert J., Wyns L. (1998). Legume lectin structure. Biochim. Biophys. Acta (Bba) Protein Struct. Mol. Enzymol..

[111] Naithani S., Komath S.S., Nonomura A., Govindjee G. (2021). Plant lectins and their many roles: Carbohydrate-binding and beyond. J. Plant Physiol..

[112] Davis A., Atwood J.L. (2017). Synthetic Lectins. Comprehensive Supramolecular Chemistry II.

[113] Davis A.P. (2010). Sticking to sugars. Nature.

[114] Ferrand Y., Crump M.P., Davis A.P. (2007). A Synthetic Lectin Analog for Biomimetic Disaccharide Recognition. Science.

[115] Liu W., Tan Y., Jones L.O., Song B., Guo Q.H., Zhang L., Qiu Y., Feng Y., Chen X.Y., Schatz G.C. (2021). PCage: Fluorescent Molecular Temples for Binding Sugars in Water. J. Am. Chem. Soc..

[116] Amrhein F., Mazik M. (2021). Compounds Combining a Macrocyclic Building Block and Flexible Side-Arms as Carbohydrate Receptors: Syntheses and Structure-Binding Activity Relationship Studies. Eur. J. Org. Chem..

[117] Yang W., He H., Drueckhammer D.G. (2001). Computer-Guided Design in Molecular Recognition: Design and Synthesis of a Glucopyranose Receptor. Angew. Chem. Int. Ed..

[118] Furikado Y., Nagahata T., Okamoto T., Sugaya T., Iwatsuki S., Inamo M., Takagi H.D., Odani A., Ishihara K. (2014). Universal Reaction Mechanism of Boronic Acids with Diols in Aqueous Solution: Kinetics and the Basic Concept of a Conditional Formation Constant. Chem. A Eur. J..

[119] Brooks W.L.A., Sumerlin B.S. (2016). Synthesis and Applications of Boronic Acid-Containing Polymers: From Materials to Medicine. Chem. Rev..

[120] Liu Z., He H. (2017). Synthesis and Applications of Boronate Affinity Materials: From Class Selectivity to Biomimetic Specificity. Acc. Chem. Res..

[121] Rowan A.E., Rowan S.J., Aida T., James T.D., Phillips M.D., Shinkai S. (2006). Boronic Acids in Saccharide Recognition.

[122] Yamashina M., Akita M., Hasegawa T., Hayashi S., Yoshizawa M. (2017). A polyaromatic nanocapsule as a sucrose receptor in water. Sci. Adv..

[123] James T.D., Sandanayake K.R.A.S., Shinkai S. (1994). A Glucose-Selective Molecular Fluorescence Sensor. Angew. Chem. Int. Ed. Engl..

[124] Wang F., Lu M., Yuan H., Zhang Y., Ji W., Sun C., Peng W. (2021). pM Level and Large Dynamic Range Glucose Detection Based on a Sandwich Type Plasmonic Fiber Sensor. J. Light. Technol..

[125] Chen Q., Fu Y., Zhang W., Ye S., Zhang H., Xie F., Gong L., Wei Z., Jin H., Chen J. (2017). Highly sensitive detection of glucose: A quantitative approach employing nanorods assembled plasmonic substrate. Talanta.

[126] Kajisa T., Sakata T. (2014). Fundamental Properties of Phenylboronic-Acid-Coated Gate Field-Effect Transistor for Saccharide Sensing. ChemElectroChem.

[127] Tlili C., Badhulika S., Tran T.T., Lee I., Mulchandani A. (2014). Affinity chemiresistor sensor for sugars. Talanta.

[128] Yang K.A., Barbu M., Halim M., Pallavi P., Kim B., Kolpashchikov D.M., Pecic S., Taylor S., Worgall T.S., Stojanovic M.N. (2014). Recognition and sensing of low-epitope targets via ternary complexes with oligonucleotides and synthetic receptors. Nat. Chem..

[129] Nakatsuka N., Yang K.A., Abendroth J.M., Cheung K.M., Xu X., Yang H., Zhao C., Zhu B., Rim Y.S., Yang Y. (2018). Aptamer-field-effect transistors overcome Debye length limitations for small-molecule sensing. Science.

[130] Sookcharoenpinyo B., Klein E., Ferrand Y., Walker D.B., Brotherhood P.R., Ke C., Crump M.P., Davis A.P. (2012). High-Affinity Disaccharide Binding by Tricyclic Synthetic Lectins. Angew. Chem. Int. Ed..

[131] Yan J., Springsteen G., Deeter S., Wang B. (2004). The relationship among pKa, pH, and binding constants in the interactions between boronic acids and diols—it is not as simple as it appears. Tetrahedron.

[132] Peters J.A. (2014). Interactions between boric acid derivatives and saccharides in aqueous media: Structures and stabilities of resulting esters. Coord. Chem. Rev..

[133] Stephenson-Brown A., Wang H.C., Iqbal P., Preece J.A., Long Y., Fossey J.S., James T.D., Mendes P.M. (2013). Glucose selective Surface Plasmon Resonance-based bis-boronic acid sensor. Analyst.

[134] Bian Z., Liu A., Li Y., Fang G., Yao Q., Zhang G., Wu Z. (2020). Boronic acid sensors with double recognition sites: A review. Analyst.

[135] Qian S., Liang Y., Ma J., Zhang Y., Zhao J., Peng W. (2015). Boronic acid modified fiber optic SPR sensor and its application in saccharide detection. Sens. Actuators Chem..

[136] Yuan H., Ji W., Chu S., Qian S., Wang F., Masson J.F., Han X., Peng W. (2018). Fiber-optic surface plasmon resonance glucose sensor enhanced with phenylboronic acid modified Au nanoparticles. Biosens. Bioelectron..

[137] Larkin J.D., Frimat K.A., Fyles T.M., Flower S.E., James T.D. (2010). Boronic acid based photoinduced electron transfer (PET) fluorescence sensors for saccharides. New J. Chem..

[138] Mou Q., Xue X., Ma Y., Banik M., Garcia V., Guo W., Wang J., Song T., Chen L.Q., Lu Y. (2022). Efficient delivery of a DNA aptamer-based biosensor into plant cells for glucose sensing through thiol-mediated uptake. Sci. Adv..

[139] Sandanayake K.R.A.S., Nakashima K., Shinkai S. (1994). Specific recognition of disaccharides by trans-3,3’-stilbenediboronic acid: Rigidification and fluoresecence enhancement of the stilbene skeleton upon formation of a sugar–stilbene macrocycle. J. Chem. Soc. Chem. Commun..

[140] Zhang X.t., Wang S., Xing G.w. (2015). Novel Boronlectins Based on Bispyridium Salt with a Flexible Linker: Discriminative Sensing of Lactose and Other Monosaccharides and Disaccharides in Aqueous Solution. Chem. Asian J..

[141] Chi L., Zhao J., James T.D. (2008). Chiral Mono Boronic Acid As Fluorescent Enantioselective Sensor for Mono *α*-Hydroxyl Carboxylic Acids. J. Org. Chem..

[142] Egawa Y., Seki T., Takahashi S., Ichi Anzai J. (2011). Electrochemical and optical sugar sensors based on phenylboronic acid and its derivatives. Mater. Sci. Eng. C.

[143] Williams G.T., Kedge J.L., Fossey J.S. (2021). Molecular Boronic Acid-Based Saccharide Sensors. ACS Sens..

[144] D’Hooge F., Elfeky S.A., Flower S.E., Pascu S.I., Jenkins A.T.A., Elsen J.M.H.v.d., James T.D., Fossey J.S. (2012). Biotinylated boronic acid fluorophore conjugates: Quencher elimination strategy for imaging and saccharide detection. RSC Adv..

[145] Pushina M., Penavic A., Farshbaf S., Anzenbacher P. (2021). Fluorescent Sensor Array for Quantitative Determination of Saccharides. ACS Sens..

[146] Seraj S., Rouhani S., Ranjbar Z., Esfahani S.L. (2021). Fructose recognition using novel solid-state electro-optical nanosensor based on boronate-tagged fluorophore modified graphene oxide. Mater. Chem. Phys..

[147] Shoji E., Freund M.S. (2002). Potentiometric Saccharide Detection Based on the pKa Changes of Poly(aniline boronic acid). J. Am. Chem. Soc..

[148] Kong K.V., Lam Z., Lau W.K.O., Leong W.K., Olivo M. (2013). A Transition Metal Carbonyl Probe for Use in a Highly Specific and Sensitive SERS-Based Assay for Glucose. J. Am. Chem. Soc..

[149] Cieplak M., Kutner W. (2016). Artificial Biosensors: How Can Molecular Imprinting Mimic Biorecognition?. Trends Biotechnol..

[150] Kajisa T., Sakata T. (2018). Molecularly Imprinted Artificial Biointerface for an Enzyme-Free Glucose Transistor. ACS Appl. Mater. Interfaces.

[151] Okutucu B., Önal S. (2011). Molecularly imprinted polymers for separation of various sugars from human urine. Talanta.

[152] Sakata T., Nishitani S., Kajisa T. (2020). Molecularly imprinted polymer-based bioelectrical interfaces with intrinsic molecular charges. RSC Adv..

[153] Kirk C., Jensen M., Kjaer C.N., Smedskjaer M.M., Larsen K.L., Wimmer R., Yu D. (2009). Aqueous batch rebinding and selectivity studies on sucrose imprinted polymers. Biosens. Bioelectron..

[154] An J.Y., Azizov S., Kumar A.P., Lee Y.I. (2018). Quantitative Analysis of Artificial Sweeteners by Capillary Electrophoresis with a Dual-Capillary Design of Molecularly Imprinted Solid-Phase Extractor. Bull. Korean Chem. Soc..

[155] Mugo S.M., Alberkant J. (2020). Flexible molecularly imprinted electrochemical sensor for cortisol monitoring in sweat. Anal. Bioanal. Chem..

[156] Doué M., Bichon E., Dervilly-Pinel G., Pichon V., Chapuis-Hugon F., Lesellier E., West C., Monteau F., Le Bizec B. (2012). Molecularly imprinted polymer applied to the selective isolation of urinary steroid hormones: An efficient tool in the control of natural steroid hormones abuse in cattle. J. Chromatogr. A.

[157] Culver H.R., Peppas N.A. (2017). Protein-Imprinted Polymers: The Shape of Things to Come?. Chem. Mater..

[158] Sullivan M.V., Dennison S.R., Archontis G., Reddy S.M., Hayes J.M. (2019). Toward Rational Design of Selective Molecularly Imprinted Polymers (MIPs) for Proteins: Computational and Experimental Studies of Acrylamide Based Polymers for Myoglobin. J. Phys. Chem. B.

[159] Selvolini G., Marrazza G. (2017). MIP-Based Sensors: Promising New Tools for Cancer Biomarker Determination. Sensors.

[160] Zarycz M.N.C., Fonseca Guerra C. (2018). NMR 1H-Shielding Constants of Hydrogen-Bond Donor Reflect Manifestation of the Pauli Principle. J. Phys. Chem. Lett..

[161] Piletska E.V., Guerreiro A.R., Romero-Guerra M., Chianella I., Turner A.P., Piletsky S.A. (2008). Design of molecular imprinted polymers compatible with aqueous environment. Anal. Chim. Acta.

[162] Park R., Jeon S., Jeong J., Park S.Y., Han D.W., Hong S.W. (2022). Recent Advances of Point-of-Care Devices Integrated with Molecularly Imprinted Polymers-Based Biosensors: From Biomolecule Sensing Design to Intraoral Fluid Testing. Biosensors.

